# Bilateral SERS‐Microneedle Patch for Co‐Diagnosis of Diabetes Mellitus and Tuberculosis Comorbidity

**DOI:** 10.1002/advs.75722

**Published:** 2026-05-25

**Authors:** Xueqin Huang, Lingzhi Chen, Jing Xu, Jiaqi Yu, Shanze Chen, Huaihong Cai, Yanguang Cong, Pinghua Sun, Jiang Pi, Lang Rao, Jierong Chen, Junxia Zheng, Haibo Zhou

**Affiliations:** ^1^ The First Dongguan Affiliated Hospital Guangdong Provincial Key Laboratory of Medical Immunology and Molecular Diagnostics School of Medical Technology Guangdong Medical University Dongguan China; ^2^ State Key Laboratory of Bioactive Molecules and Druggability Assessment Guangdong Basic Research Center of Excellence for Natural Bioactive Molecules and Discovery of Innovative Drugs The Fifth Affiliated Hospital College of Pharmacy Jinan University Guangzhou China; ^3^ College of Chemistry and Materials Science Jinan University Guangzhou China; ^4^ Institute for Safflower Industry Research Key Laboratory of Xinjiang Phytomedicine Resource and Utilization Ministry of Education School of Pharmacy Shihezi University Shihezi China; ^5^ Institute of Chemical Biology Shenzhen Bay Laboratory Shenzhen China; ^6^ Department of Clinical Laboratory Guangdong Provincial People's Hospital (Guangdong Academy of Medical Sciences) Southern Medical University Guangzhou Guangdong China; ^7^ School of Biomedical and Pharmaceutical Sciences Guangdong University of Technology Guangzhou China

**Keywords:** diabetes mellitus, microneedle, surface‐enhanced raman scattering, tuberculosis

## Abstract

The prevalence of diabetes mellitus (DM) and tuberculosis (TB) comorbidity is constantly rising worldwide, thus there is an urgent need for a co‐diagnostic method of TB‐DM in the clinic. Here, we introduced a bilateral surface‐enhanced Raman scattering (SERS)‐microneedle (MN) patch that incorporated Au hybrid mesoporous polydopamine‐coated urchin Au/Ag (U@mP@Au) for analyzing glucose in interstitial fluid (ISF) and TB biomarker (ESAT‐6/CFP‐10) in sputum toward the co‐detection of TB‐DM comorbidity. The feasibility of the bilateral SERS‐MN patch was demonstrated in ex vivo artificial skin, in vivo mouse models, and trials of recruiting patients. In the clinical assessment, DM patients (*N = 18*) and healthy subjects (*N = 3*) were classified with satisfactory sensitivity (88.9%) and specificity (100%), among which one DM positive sample (P11) with TB infection was successfully identified. Results revealed the sensitivity (87.5%) and specificity (90.9%) of SERS‐MN array for TB diagnosis *(N = 33*), which were comparable to traditional clinical methods (smear, TST, and Xpert). More importantly, three TB‐positive patients (P9, P15, and P20) showed abnormally elevated glucose levels, which is highly suggestive of TB‐DM complication. This integrated dual‐analyte detecting platform represented a significant step toward the cooperative diagnosis of TB‐DM comorbidity, facilitating the early detection of complications by clinicians.

## Graphical abstract

Our study introduced a U@mP@Au‐mediated bilateral SERS‐microneedle patch for analyzing glucose levels from ISF and sensitively detecting TB biomarkers from sputum, respectively. This integrated dual‐analyte detecting platform can be used for co‐diagnosis of diabetes mellitus (DM) and tuberculosis (TB) comorbidity, which was demonstrated in ex vivo artificial skin, in vivo mouse models, and trials of recruiting patients.

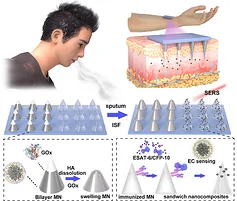

## 1 Introduction

Tuberculosis (TB) is one of the major global public health issues, despite continuous efforts to prevent, control, and treat it [1]. Diabetes mellitus (DM), a common metabolic disease, has become a major threat to TB control [2]. The coexistence of DM and TB (TB‐DM) has important health consequences as DM significantly increases susceptibility to TB infection and deteriorates treatment outcomes. At present, the number of deaths caused by TB‐DM comorbidity accounts for approximately 11% of the total deaths of TB patients worldwide [3]. Therefore, the synergetic diagnosis of TB‐DM comorbidity is of paramount significance for shortening the treatment time, improving the treatment efficiency, and reducing recurrence risk in TB patients. However, current studies only conduct separate tests for TB infection or DM [4, 5, 6, 7], lacking the collaborative testing capabilities.

In the clinic, blood glucose levels are an important indicator for DM diagnosis, but the blood extraction is painful and inconvenient for the patients. Recently, microneedle (MN) with hundreds of tiny tips (50–600 µm) can bypass the nerve and vessel located in the deeper dermis to monitor subcutaneous glucose levels in interstitial fluid (ISF) [8], featuring minimal invasiveness, painlessness, good tolerance, and easy operation [9]. According to the analytic purposes, a variety of MN‐based strategies have been developed to monitor biomarkers in ISF, including suction‐based ISF extraction through microporous MN, diffusion‐based ISF collection through swell MN, and immuno‐functionalized MN for ISF interaction and subsequent on‐needle analysis [10, 11, 12, 13]. Nowadays, with the escalating demand of the people for measuring their health conditions, the application of MN has also been expanded and is no longer limited to ISF extraction and detection. Through the modification of biorecognition units (e.g., antibodies, peptides, and aptamers), MN can specifically capture various biomarkers from body fluids (e.g., saliva, sputum, and blood), and then conduct ex vivo assays [14, 15]. This method enables MN to overcome the major concern of limited biomarkers in ISF, and is applicable to a wider range of disease detection. However, all these MN‐based systems require highly sensitive techniques to amplify and transmit the detection signals, due to the low biomarker content in fluid biopsy, particularly for ISF, where the biomarkers are only present at the ≈fg/mL level.

To tackle the aforementioned issues, surface‐enhanced Raman spectroscopy (SERS) stands out for its high sensitivity for the analysis of biomarkers by utilizing the electromagnetic “hotspots” of plasmonic nanostructures (e.g., Au, Ag, and Cu) [16, 17, 18]. This SERS technique also provides molecular fingerprint information of low‐concentration analytes and is widely used for the chemical analysis of metabolites, ions, proteins, and other components in body fluids. Recently, many studies integrated a wearable MN patch with highly sensitive SERS sensors for direct in situ intradermal measurements [19, 20, 21]. Our group has previously reported the development of a flexible MN‐based SERS sensor for early screening of melanoma [22]. However, the SERS performance largely depends on the size and geometry of plasmonic nanostructures. Therefore, how to develop an appropriate SERS substrate assembly with MN for highly sensitive detection of body fluids remains a huge challenge.

**SCHEME 1:**
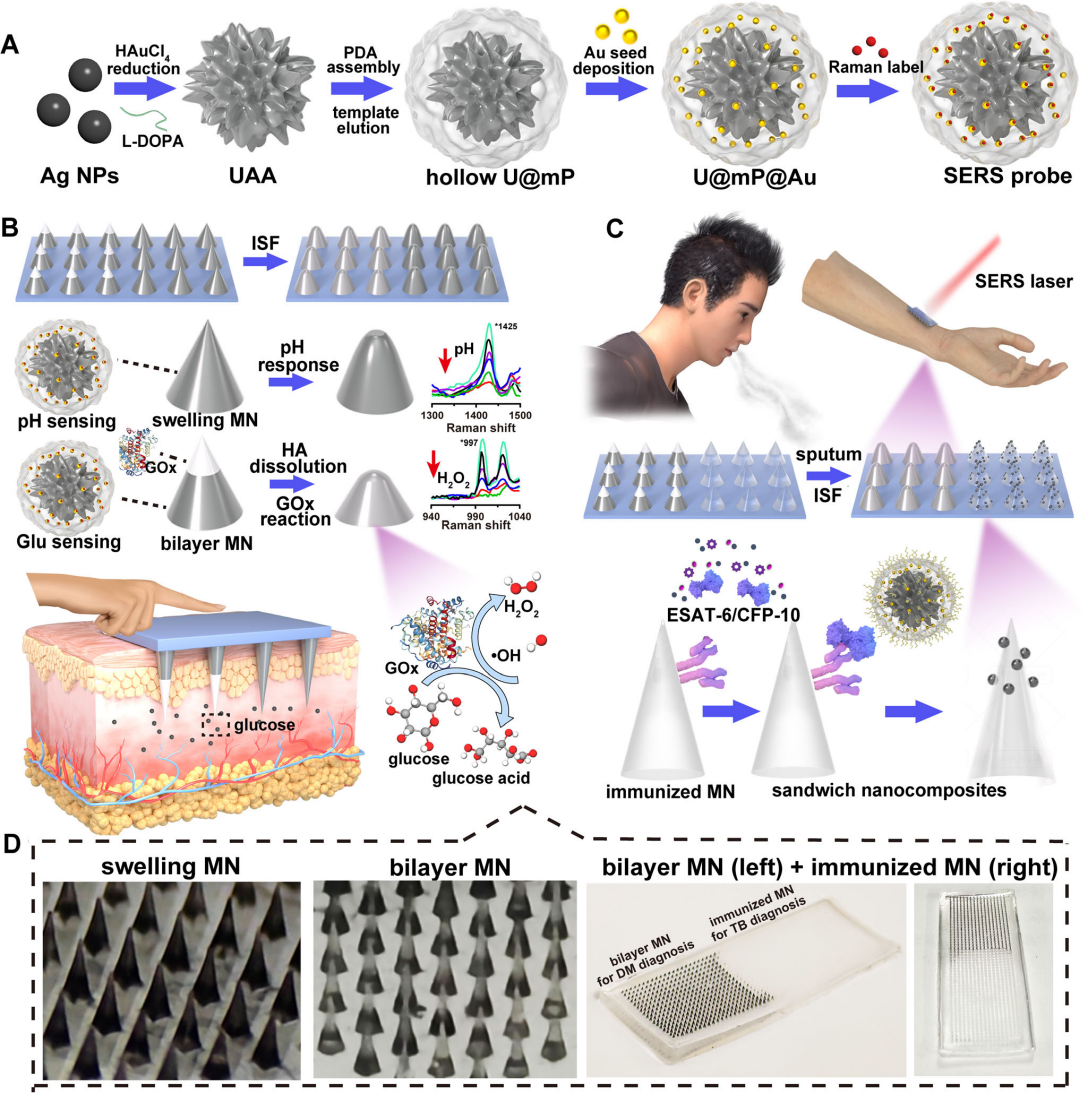
(A) Schematic illustration of the fabrication process for U@mP@Au (SERS probe). (B) Principle of bilateral SERS‐MN patch made in bilayer MN and swelling MN for detection of glucose and pH. (C) Principle of bilateral SERS‐MN patch made in bilayer MN and immunized MN for co‐diagnosis of TB‐DM comorbidity. (D) Optical images of different types of MN.

In this work, we fabricated a core‐satellite nanostructure of U@mP@Au that was incorporated with a bilateral MN patch for the analysis of glucose in cutaneous ISF, and *Mycobacterium tuberculosis* (*Mtb*) specific ESAT‐6/CFP‐10 antigen in sputum toward collaborative detection of TB‐DM comorbidity. U@mP@Au was composed of urchin‐shaped Au/Ag (UAA) core, mesoporous polydopamine (mPDA) shell, and external Au nanoparticles (NPs) that emitted strong, stable, and repeatable SERS signals for ultrasensitive and quantitative monitoring of various biomarkers in ISF or sputum (Scheme 1A). Two strategies were respectively designed for the bilateral MN patch (Scheme 1C): one side was bilayer MN, where the upper layer was made of soluble Hyaluronic Acid (HA), which was entrapped with glucose oxidase (GOx). The upper layer could rapidly dissolve when inserted into the skin to catalyze glucose into hydrogen peroxide (H_2_O_2_), and subsequently absorbed by the lower layer made of swellable methacrylate hyaluronic acid (MeHA) that incorporated U@mP@Au for SERS sensing of glucose; and the other side was antibody (Ab)‐modified insoluble MN for capturing the ESAT‐6/CFP‐10 antigen from sputum, followed by on‐needle SERS analysis with the immuno‐assisted of U@mP@Au to assess TB infection. Furthermore, considering the significance of pH measurement during bacterial infection, we developed another MeHA‐based swell SERS‐MN system containing U@mP@Au for pH monitoring in ISF (Scheme 1B). Therefore, unlike the current technologies that can only detect DM or TB infection in a separate way, this integrated dual‐analyte detecting platform not only analyzes glucose and pH levels from ISF but also sensitively detects TB biomarkers from sputum, holding great potential in the co‐diagnosis of TB‐DM comorbidity (Scheme 1D). Notably, this MN patch with painless, safe, and minimally invasive characteristics substantially improved patient compliance.

## 2 Results and Discussion

### 2.1 The Formation Process and Mechanism of U@mP@Au

**FIGURE 1:**
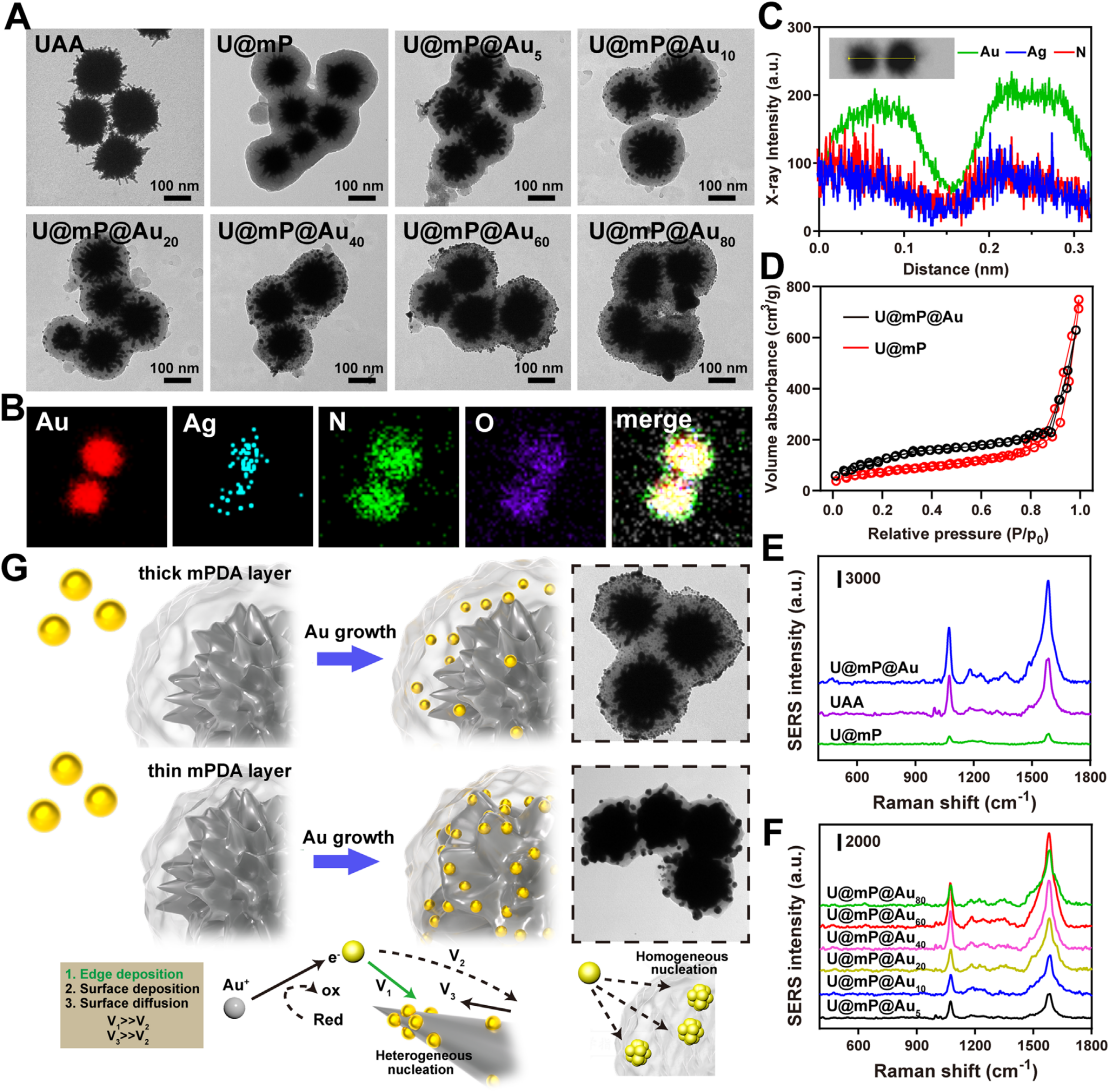
Synthesis and characterization of U@mP@Au. (A) TEM images of UAA, U@mP, U@mP@Au_5_, U@mP@Au_10_, U@mP@Au_20_, U@mP@Au_40_, U@mP@Au_60_, and U@mP@Au_80_. (B) EDS elemental mapping of Au, Ag, N, and O from U@mP@Au. (C) The line scan of Au, Ag, and N from U@mP@Au. (D) N_2_ adsorption‐desorption isotherms curve of U@mP and U@mP@Au, (E) SERS spectra of UAA, U@mP and U@mP@Au. (F) SERS spectra of U@mP@Au prepared with different contents of HAuCl_4_ solution. (G) Schematic illustration for homogeneous or heterogeneous nucleation of Au nanocrystals in different thicknesses of mPDA shell.

Herein, Au hybrid mesoporous polydopamine‐coated urchin Au/Ag (U@mP@Au) was developed as SERS probe with a large Raman cross‐section for intense SERS enhancement. The synthesis process was depicted in Scheme 1A. Initially, urchin‐shaped Au/Ag alloy (UAA) was fabricated through the seed‐mediated growth and in situ reduction reactions, in which the typical sharp tentacles and large surface areas endowed them with the intense electromagnetic field intensity and distribution. Then, mPDA was grafted on the surface of UAA core based on the self‐polymerization of DA under alkaline conditions, followed by removing the template to form the dense mesoporous structure. The mPDA shell as a protective layer can maintain the stability and monodispersity of SERS probes, and its large pores facilitate the mass transfer of substances. Finally, Au NPs were embedded into mPDA shell through in situ growth to obtain the multi‐compartment structure of U@mP@Au. Transmission electron microscopy (TEM) showed the open uniform mesopores on the regular spherical shape of PDA surface, while the reduction of Au seed made the surface of mPDA rough and bulging (Figure 1A). Furthermore, the energy‐dispersive X‐ray (EDX) plot of U@mP@Au evidently displayed the chemical distribution of Au, Ag, N, and O over the whole NPs (Figure 1B). The line scan profile across the region in Figure 1C further indicated the external shell layer dotted with Au seed. The Au interspersed U@mP core/satellite structure had good uniformity from batch to batch (Figure S1).

The structural integrity of U@mP@Au was evaluated through X‐ray diffraction (XRD) analysis (Figure S2). The presence of strong peaks at Bragg angles (2θ) = 38.2°, 44.5°, 64.7°, and 77.8° was indexed to the (111), (200), (220), and (311) lattice planes of Au/Ag, respectively, which suggested the existence of Au/Ag alloys. However, the mPDA can hardly be found by the XRD due to its amorphous nature. X‐ray photoelectron spectroscopy (XPS) was performed to analyze the surface state elemental components. The XPS spectra displayed the C1s, N1s, and O1s peaks (Figure S3), which originated from the mPDA and residue polymer component in the nanostructure. From Figure S4, the binding energies at 84.08 and 87.88 eV correspond to Au 4f_7/2_ and Au 4f_5/2_, and 374.28 and 368.38 eV correspond to Ag 3d_3/2_ and Ag 3d_5/2_, respectively, elucidating the charge transfer of Au and Ag in the nanostructure and the formation of Au/Ag alloy. Brunauer–Emmett–Teller (BET) analysis was further investigated to investigate the mesoporous structure of U@mP following Au deposition. The nitrogen adsorption‐desorption isotherms of U@mP and U@mP@Au showed the typical type IV isotherm according to the IUPAC nomenclature (Figure 1D), while Au coverage slightly reduced the pore size and specific surface area.

The resonance properties were closely related to the changes in structure; thus, the optical performance of UAA, U@mP, and U@mP@Au was further assessed. As illustrated in Figure 1E, when the mPDA shell was assembled on the surface of UAA core, a significant decrease in SERS intensity was observed owing to the coverage of mPDA layer for plasmonic hotspots on substrates. However, SERS signals were recovered and enhanced for several orders of magnitude, once the Au NPs were densely distributed on the interlayer of as‐formed mPDA shell for highly aggregated plasmonic hotspots, with the enhancement factor (EF) of 4.52 × 10^8^. Furthermore, the density of Au nanocrystals in the U@mP@Au was mainly dictated by the amount of HAuCl_4_ in the reaction system. Au NPs formed scattered nanoclusters within the mPDA framework at the low content (HAuCl_4_ = 5 µL), and became larger and denser with the increase of HAuCl_4_ content (Figure 1A). SERS responses also increased with the increase of the density of Au nanoclusters, as the abundant aggregation and agglomeration of Au enhanced the local electromagnetic field (Figure 1F). However, excessive HAuCl_4_ caused a decrease in SERS intensity, possibly because the further growth of Au thickened the Au layer on U@mP, which inevitably shielded the Raman reporters from being stimulated by the scattering light. The optimal SERS performance was U@mP@Au_60_ (HAuCl_4_ = 60 µL), which was used for further experiments (Figure S5). Furthermore, the growth time of Au nanocrystals was also explored while keeping the HAuCl_4_ concentration constant. The density of Au NPs gradually increased over time and reached saturation at 15 min (Figure S6). As a consequence, the subsequent regrowth time of Au was determined to be 15 min.

To shed light on the mechanism of forming U@mP@Au, we evaluated the effect of mPDA thickness on subsequent Au growth. For the thin mPDA layer, most Au atoms can easily penetrate the mPDA layer and deposit on the UAA surface, while only a small portion remained in the mPDA scaffolds for self‐nucleation into Au nanocrystals (Figure 1G). More interestingly, the accumulation of Au atoms facilitated the anisotropic growth of UAA, transforming the morphology of spiny sea urchins into spherical NPs. In theory, the overall growth of U@mP@Au followed two paths [23]. The homogeneous Au atoms pass through the thin layer of mPDA and randomly attach to the tips and surfaces of UAA. Since the deposition rate of Au at the edge (V1) is faster than that at the surface (V2), Au atoms are preferentially accumulated on the tips of UAA. Additionally, the atomic surface diffusion rate (V3) is greater than V2; thus, Au atoms tend to diffuse toward the edge when attached to the surface. Therefore, the tip is extensively covered with Au atoms, and subsequently, the heterogeneous proliferation of Au atoms on the surface of UAA makes the surface smoother and the morphology gradually spherical. To further confirm this conjecture, we reduced the dosage of dopamine hydrochloride by half and shortened the polymerization time to facilitate the observation of Au growth process. As expected, Au atom diffused into the mPDA layer and began to deposit preferentially at the spire (Figure S7, red circle), affirming the conjecture for Au diffusion and growth. However, a thick mPDA layer can prevent the shrouding growth of Au on UAA surface, because the hierarchical interlaced structure effectively hinders the free diffusion of HAuCl_4_, retaining it in the polymerized layer for homogeneous nucleation of Au nanostructures. Moreover, we found that when the content of UAA in the system was insufficient, mPDA not only wrapped on UAA surface, but also underwent self‐polymerization to form a clumped layer, in which Au was further reduced to uniform small and intense particles (Figure S8, red arrow). The above data elucidated the formation process and mechanism of U@mP@Au, facilitating its incorporation with MN to form SERS‐MN sensitive response platform.

### 2.2 Bilateral SERS‐MN Sensing Platform

**FIGURE 2:**
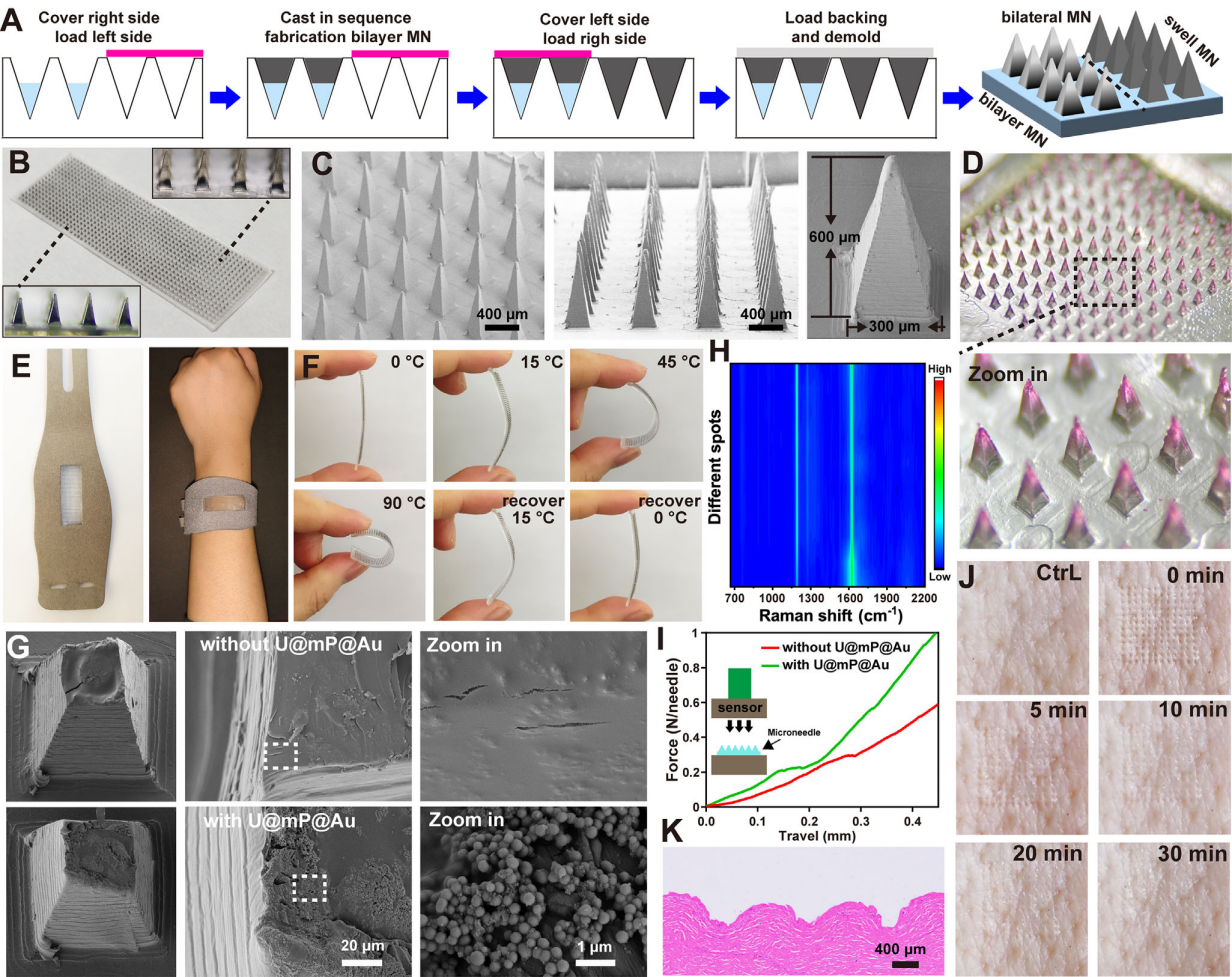
Synthesis and characterization of MN. (A) Fabrication process of the bilateral MN patch. (B) Bright field images of the bilateral MN patch, where one side was a swellable MN and the other side was bilayer MN. (C) SEM images of MN array. (D) Microscopy images illustrating the double‐layer structure of MN array. Optical image demonstrating the (E) wearability and (F) flexibility of MN patch. (G) SEM showing the distribution of U@mP@Au within the MN. (H) 2D Raman spectra for different regions in MN. (I) Mechanical properties of MN with or without U@mP@Au. (J) The porcine skin recovery process after puncturing with MN array. (K) Histological images of porcine skin after puncturing with MN array.

The detailed process for manufacturing bilateral MN through Polydimethylsiloxane (PDMS) mold was shown in Figure 2A, where one side was a swellable MN constructed from MeHA and U@mP@Au for pH sensing; another side was a bilayer MN composed of the soluble HA/GOx on the upper layer, and the swellable MeHA/U@mP@Au on the lower layer for glucose detection in ISF. Specifically, the upper layer was first poured into the mold by controlling the centrifugal speed to form a needle tip, and then the lower layer was poured in sequence to construct a double‐layer MN patch (Figure 2B). To have better visualization, we stained the upper parts with R6G and kept the original color of MeHA in the lower parts. Two distinct colors on the MN confirmed the successful construction of the bilayer structure (Figure 2D). The construction method of swellable MN was similar to that of bilayer MN, except that the upper layer HA was not included. The bilateral MN was first sealed and blocked in the PDMS model through adhesive tape, and each part of MN was cast in sequence with the assistance of centrifugal force.

To meet user demand for portability, we combined the micro‐molding techniques to design and fabricate MN with appropriate types, including thickness, size, and edges, followed by integrating them into various wearable formats, such as wristbands and arm belts (Figure 2E). It is worth noting that the prepared MN array was assembled into a patch with high flexibility, which can withstand mechanical deformation and maintain its shape well even after being bent at 90°C (Figure 2F). This stretchable and soft property, on the one hand, brought comfort to users, and on the other hand, enabled the detection of bent arms more conveniently. To characterize the fine structure of MN, we constructed the MN patch in 12 × 12 array. SEM images pointed out that each needle of the MN patch was neatly arranged with a similar morphology. The MN showed a pyramidal structure with a sharp tip of 600 µm, a base diameter of 300 µm, and a center spacing of 600 µm (Figure 2C). The appropriate length and size of MN enable it to sample ISF with minimal invasiveness and pain. Furthermore, MN has good reproducibility among different batches, which is necessary for the subsequent ISF analysis (Figure S9).

In addition to the morphology and size of MN, sufficient mechanical strength is the fundamental prerequisite for MN transdermal detection of ISF [24]. The graph of force versus displacement showed that the slope of U@mP@Au‐loaded MN was higher than that of blank MN (Figure 2I), presumably because U@mP@Au filled the sparse space of MeHA to form a dense network for enhancing the mechanical properties of MN. The insertion capability of MN patch was assessed by pressing it into the isolated porcine skin. After the removal of MN patch, discernible micro‐holes were found on the porcine skin. The skin gradually recovered within 20 min and completely returned to the original state at 30 min, exhibiting minimally invasive characteristics of MN (Figure 2J). Histological section analysis revealed that the penetration depth of MN was ∼500 µm, confirming that the tip of MN only reached the epidermis without penetrating the dermis rich in blood vessels and nerve endings (Figure 2K). The biosafety of MN array was further ascertained by the Cell Counting Kit‐8 (CCK‐8) assay (Figure S10). The results showed that the viability of HUVEC cells remained almost unchanged before and after MN immersion, indicating the biocompatibility of MN materials. Further considering the potential toxicity of leakage of metal materials, we detected the Au ions concentration on the porcine skin by ICP‐MS after MN insertion. It can be seen that there was almost no distribution of U@mP@Au on the skin, which can eliminate the biosafety issues caused by U@mP@Au detachment from MN (Figure S11). Therefore, compared with similar types of MN sensors, this MN patch is more flexible, scalable, robust, and biocompatible, with high patient compliance (Table S1).

In this study, U@mP@Au‐based MN was designed to detect pH and glucose in ISF, so whether U@mP@Au was effectively distributed within MN is crucial for SERS sensing. Therefore, we scraped off the tip of MN to further analyze the internal structure of MN. As shown in Figure 2G, a high density of U@mP@Au was deposited inside MN at a content of approximately 10 NPs/µm^2^. The enlarged image showed that the surface of U@mP@Au appeared rough and uneven due to the growth of Au atoms onto the mPDA shell, which is consistent with the TEM results observed in Figure 1A. The cross‐linked structure of MeHA provided a large surface area for U@mP@Au aggregation and interaction, where dense and strong hot spots were created for highly sensitive SERS sensing. According to the obtained SERS mapped image (Figure 2H), a uniform signal was observed in the different regions of MN, which is attributed to the homogeneous and dense distribution of U@mP@Au within the MN. Hence, this SERS‐MN sensing platform held enormous potential for further ISF transdermal detection.

### 2.3 Bilateral SERS‐MN for ISF Transdermal Detection of pH/Glucose

**FIGURE 3:**
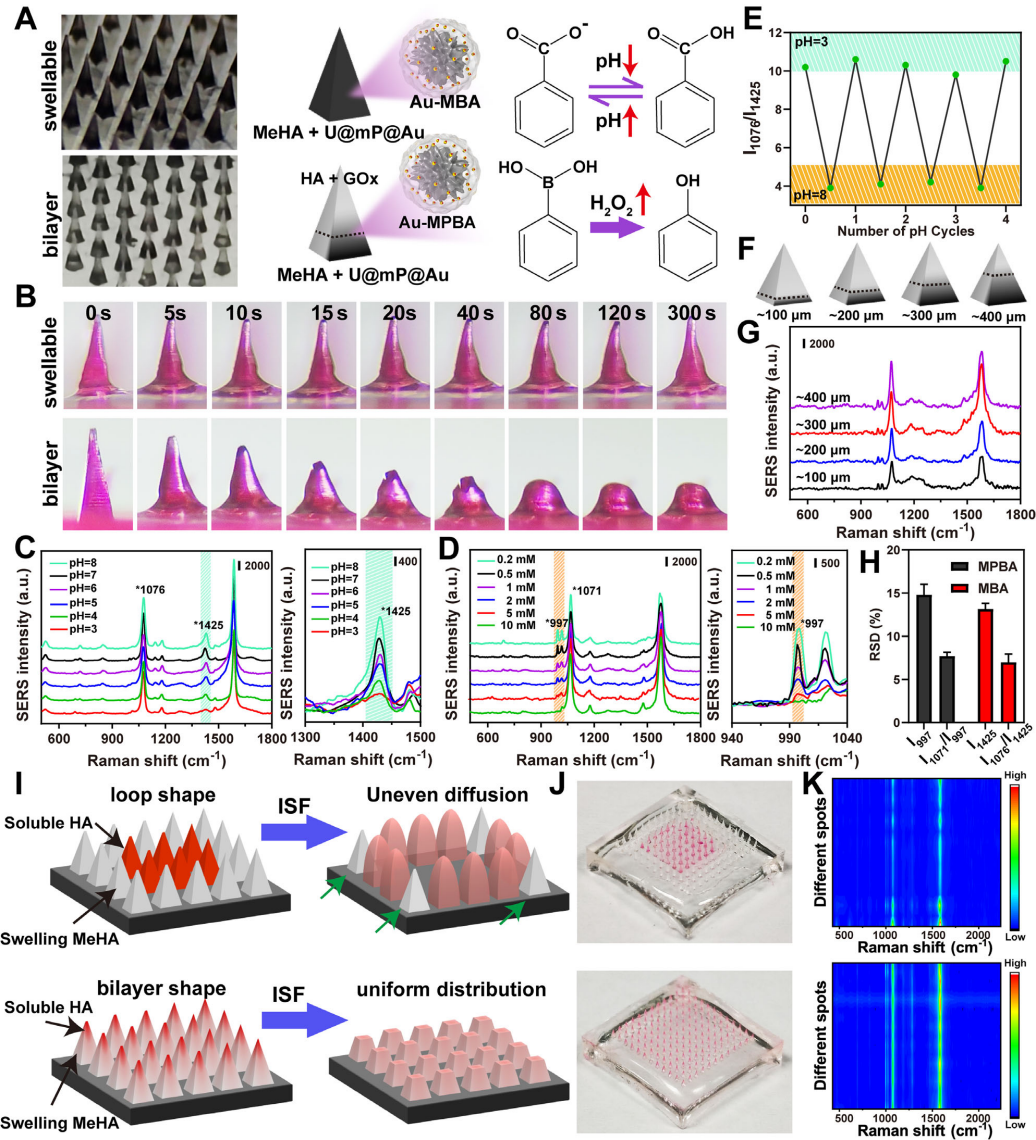
SERS‐MN for pH/glucose sensing in vitro. (A) Microscopy images of swellable or bilayer MN, and the principle of pH/glucose detection by SERS‐MN sensing. (B) Microscopy images of swellable or bilayer MN after adding PBS. (C) SERS spectra of 4‐MBA on the swellable MN array under different pH range. (D) SERS spectra of 4‐MPBA on the bilayer MN array under a range of different glucose concentrations. (E) Measurement of the cycling pH on the swellable MN array. (F) The height of lower layer in the bilayer MN. (G) SERS sensing at different heights of the lower layer. (H) RSDs (%) calculated from Raman peak intensity with and without internal standard normalization. Data are presented as mean ± SD. (*n = 3*). (I) Workflow of loop shape and bilayer shape MN when in contact with ISF. The green arrow represented MeHA MN cannot come into contact with the dissolved HA due to the long distance. (J) Bright field images of loop shape and bilayer shape MN, where R6G staining the soluble HA. (K) The corresponding 2D Raman spectra obtained from loop shape and bilayer shape MN.

For pH measurement, 4‐mercaptobenzoic acid (4‐MBA) as a pH‐responsive Raman reporter was labeled on the U@mP@Au (Figure 3A). U@mP@Au‐filled swellable MN was incubated in different pH solutions from 3 to 8, and the SERS spectra of 4‐MBA were acquired three times and averaged for detection. The characteristic SERS peak that is most responsive to pH variation commonly occurs at 1425 cm^−1^, which belongs to its symmetrical carboxylate vibration. In contrast, the peak at 1076 cm^−1^ has the minimal response to pH fluctuation and therefore is often regarded as the reference peak (Figure 3C). Relative standard deviation (RSD, %) demonstrated that the ratio of 1076 and 1425 cm^−1^ (I_1076/1425_) was more stable than the band at 1425 cm^−1^ (I_1425_) for pH analysis (Figure 3H). To further explore the reversibility of pH detection, we collected the SERS spectra of swellable MN cycle between pH values of 3 and 8. The results showed that the SERS signal changes were similar in five cycles (Figure 3E), indicating the feasibility of swellable MN for pH monitoring in ISF.

For glucose measurement, the principle is based on the GOx catalyzing ISF glucose to produce H_2_O_2_, which converts Raman reporter 4‐mercaptophenylboronic acid (4‐MPBA) modified on U@mP@Au to 4‐mercaptophenol (4‐MPhOH), causing a SERS spectral change [25]. We first loaded GOx into the tip of soluble MN, which dissolved and released when in contact with ISF (Figure 3A). H_2_O_2_ produced by GOx catalysis was absorbed by the lower layer of swellable MN, from which glucose was detected by SERS response to H_2_O_2_. Figure 3D illustrates the SERS spectra of 4‐MPBA, where the characteristic bands responsive to H_2_O_2_ at 997 cm^−1^ was attributed to C─C in‐plane bending. Compared to an unresponsive mode at 1071 cm^−1^, this H_2_O_2_‐responsive peak robustly decreased with the increase of glucose level. Similarly, to improve the accuracy of SERS detection, the Raman intensity ratio of 1071 and 997 cm^−1^ was calculated. As shown in Figure 3H, the RSD value was prominently decreased from 14.8 (I_997_) to 7.7% (I_1071/997_) after introducing an internal reference for signal calibration, thereby confirming the reliability of this SERS‐MN for glucose measurement in ISF.

To further verify this strategy, the swelling capacity of MN was determined by testing the mass change of MN patch at different times after adding PBS. As shown in Figure 3B, swellable MN fabricated by MeHA expanded rapidly within 20s and then gradually reached to stability, with significant morphological change before and after imbibition. It is reasonable because the cross‐linked MeHA was like a dry sponge that can quickly absorb moisture within a few minutes. Although MeHA swelled obviously, the morphology was well preserved due to its good mechanical strength. Unlike insoluble yet highly swellable MeHA, HA with high water affinity is usually used to make dissolvable MN for transdermal drug delivery. During the glucose detection, only when the GOx‐loaded HA tip dissolves can it react with the glucose in ISF and be further absorbed by the lower MeHA to trigger SERS sensing. Therefore, we further ascertained the dissolution and absorption efficiency of the bilayer MN constructed by the upper HA and the lower MeHA. It can be seen that HA dissolved gradually within 40 s after adding PBS, which was rapidly absorbed by the lower layer at 80 s. The lower layer swelled and maintained its morphology without changing over time, further verifying the feasibility of bilayer MN for glucose detection.

To maximize the SERS detection, we manipulated the height ratio of the upper layer and lower layer of bilayer MN (Figure 3F). As expected, the thicker the lower layer, the more accumulated U@mP@Au SERS probes can amplify the SERS signal and improve detection sensitivity (Figure 3G). However, the excessively high proportion of the lower layer, on the one hand, will reduce the GOx content in the upper layer, hampering the full catalysis of glucose; On the other hand, it will cause uneven distribution of a large amount of U@mP@Au, and different particle densities in various regions are not conducive to the stable output of SERS signals. Thus, ∼300 µm was selected as the optimal height of the bottom layer. We also optimized the content of GOx in the upper layer, and confirmed that 10 mm GOx was sufficient to complete the catalytic reaction of glucose (Figure S12). In addition, the enzymatic catalytic process was vigorous within the first 10 min, but tended to stabilize after 20 min (Figure S13). However, considering the demand for rapid screening in practical applications, a reaction time of 15 min was selected for the following experiments, by which the enzymatic catalytic process has been completed by approximately 85%. Furthermore, critical experimental factors were optimized, and results revealed that 10^−4^ m of Raman reporter can obtain the optimal SERS performance for pH/glucose sensing (Figures S14 and S15).

To underscore the advantages of bilayer MN, we further compared it with the loop shape MN, where soluble HA containing GOx was arranged in the inner circle, and swellable MeHA was designed in the outer circle (Figure 3I,J). HA dissolved and diffused in different directions when in contact with PBS solution, followed by absorption by the exterior MeHA for SERS sensing. We found out that the diffusion of HA was nondirectional, which means that only the part of MeHA close to HA can absorb the GOx released from HA (see the green arrow in Figure 3I), which will lead to the unstable distribution of SERS signals with false positive/negative results. On the contrary, the bilayer MN can tackle this issue because the dissolved upper layer has full contact with the lower layer. A shorter liquid flow distance is conducive to improving the stability of SERS detection. The SERS imaging confirmed our conjecture, where SERS signal obtained from each needle tip of the bilayer MN is more uniform and stronger than that of the loop‐shaped MN (*n* = 30) (Figure 3K). Therefore, this experiment proved the applicability of bilayer SERS‐MN for glucose detection in ISF.

### 2.4 Bilateral SERS‐MN for Glucose Analysis in ISF

**FIGURE 4:**
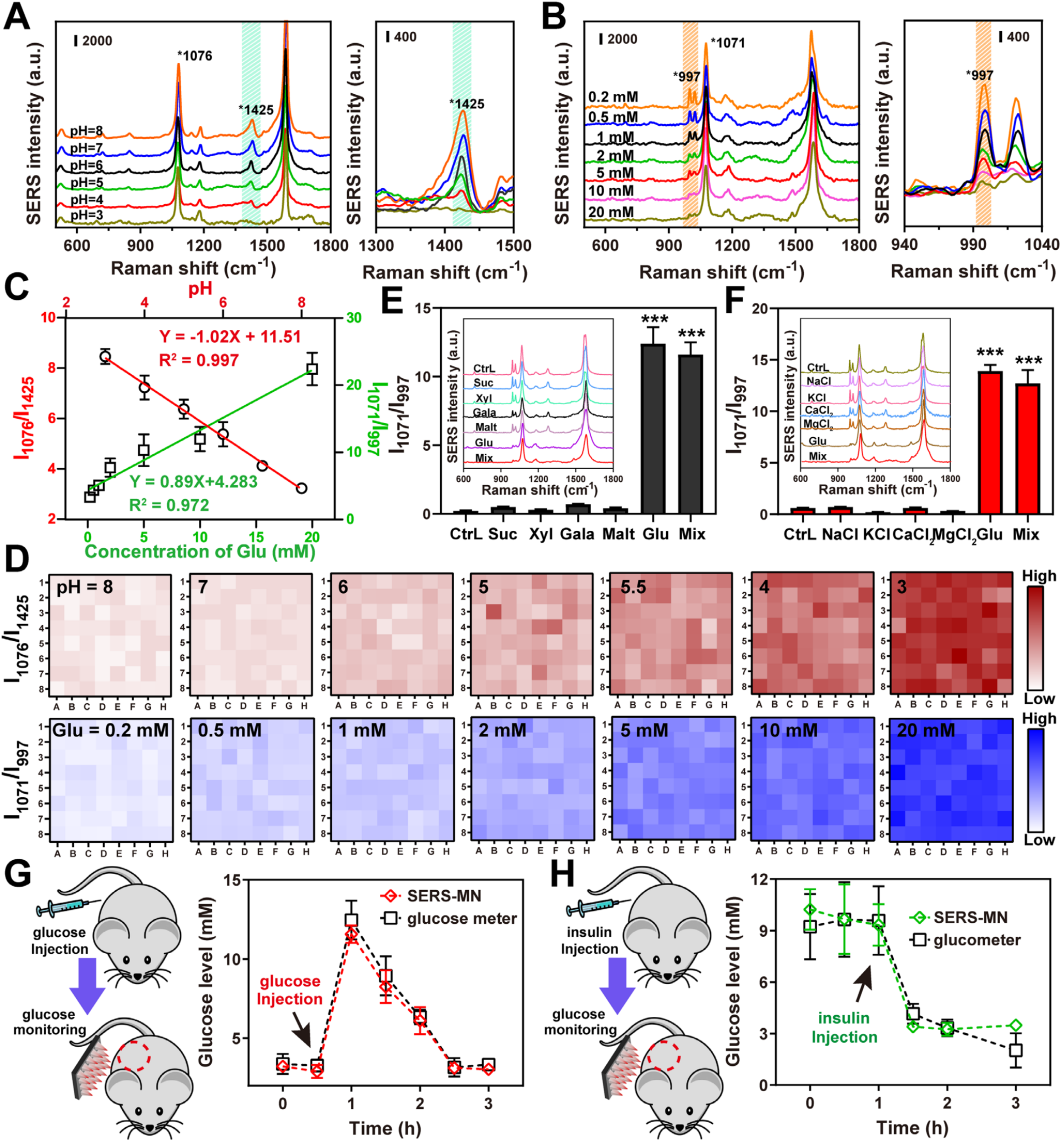
SERS‐MN for pH/glucose detection ex vivo and in vivo. SERS response of MN array in gel skin phantom with (A) different pH, and (B) glucose concentrations. (C) Standard curves of the SERS peak ratios against the glucose concentration or pH. Data are presented as mean ± SD. (*n = 3*). (D) SERS mapping images of different sides of MN were taken using the characteristic peak ratio. (E) Specificity assay of bilayer SERS‐MN. Mix = glucose (Glu) + Suc + Xyl + Gala + Malt. Inset: SERS spectra for each group. Data are presented as mean ± SD. (*n = 3*). ^***^ p < 0.001. (F) Anti‐interference assay of bilayer SERS‐MN. Mix = glucose (Glu) + NaCl + KCl + CaCl + MgCl_2_. Inset: SERS spectra for each group. Data are presented as mean ± SD. (*n = 3*). ^***^ p < 0.001. (G) The ISF glucose level of mice after injection with glucose (2 g/kg). Data are presented as mean ± SD. (*n = 3*). (H) The ISF glucose level of diabetic mice after injection with insulin (2 U/kg). Data are presented as mean ± SD. (*n* = 3).

Based on the favorable performance in vitro, bilateral SERS‐MN was further assessed in an agar gel skin phantom, which was pre‐incubated with pH and glucose to simulate the real skin ISF. Then, bilateral SERS‐MN was pressed into agar gel skin phantom, where one side is a swellable MN with 4‐MBA for recording pH, and another side is a bilayer MN with 4‐MPBA for monitoring glucose. For pH detection, the 4‐MBA SERS spectra were collected with varying pH levels from 3 to 8. Similar to the observation in Figure 3C, the pH‐responsive characteristic peak at 1425 cm^−1^ increased with pH rising, while the peak at 1076 cm^−1^ that is faintly responsive to pH was used as reference (Figure 4A). By plotting the pH value vs Raman intensity ratio of *I*_1076_ */I*_1425_, a reliable calibration curve was obtained, with a correlation coefficient of R^2^ = 0.997 (Figure 4C). For glucose measurement, H_2_O_2_ produced by GOx catalysis on the tip was absorbed by swelling MeHA on the bottom, inducing the SERS change of MPBA at 997 cm^−1^ (Figure 4B). With the increase of glucose concentration, the ratio of *I*_1071_ */I*_997_ gradually increased and exhibited a linear relationship within the range of 0.2–20 mm (R^2^ = 0.972) (Figure 4C). This glucose testing range was relatively wide, allowing it can meet the DM detection requirements in the clinic, especially suitable for patients with diabetic complications who have abnormally high levels of blood glucose. The SERS mapped profile presented a uniform and linear signal output on account of evenly distribution of U@mP@Au inside the whole region of MN (Figure 4D), where the SERS signal on MN changed accordingly with pH and glucose. Therefore, as a proof‐of‐concept demonstration, our proposed bilateral SERS‐MN displayed measurable responses to pH and glucose in the ISF with exceptional accuracy and sensitivity. However, since many respiratory infectious diseases can also induce changes in pH, the guiding effect of pH on TB‐DM comorbidity is extraordinarily weak. Therefore, in this study, we only explored the possible application of this MN in pH measurement, and further conducted in‐depth research on the detection of glucose and TB markers.

To determine the specificity of this SERS‐MN for glucose detection, the control experiments were conducted using a series of interfering substances, including sucrose (Suc), xylose (Xyl), galactose (Gala), and maltose (Malt), each at a concentration of 1 mm. As depicted in Figure 4E, the signal ratios obtained from different interfering substances were almost identical to the blank group, relative to the strong SERS intensity ratio (*I*_1071_ */I*_997_) for glucose under identical experimental conditions. Furthermore, the coexistence of interfering substances with glucose exhibited a negligible impact on the original SERS signal ratio, mainly due to the selectivity of GOx cascade catalytic strategy, in which GOx has a low ability to catalyze other sugars relatively. Given that the complex biological environments may interfere with the ISF glucose detection, diverse biologically relevant interferents, such as NaCl, KCl, CaCl_2_, and MgCl_2_, were also included as typical controls. As illustrated in Figure 4F, only in the presence of glucose can SERS signal ratios be augmented, while the SERS ratios of other species had no significant difference from the blank group. Besides, the mixture of diverse biologically relevant species presented negligible influence on glucose detection, which further highlighted the strong anti‐interference of the SERS‐MN. Furthermore, the stability of SERS‐MN was another vital parameter for practical use. As found in Figure S16, the bilayer SERS‐MN maintained a strong SERS signal ratio for glucose detection even after 20 days of storage. Overall, these results revealed that our SERS‐MN possessed exceptional selectivity, robust anti‐interference, and great stability, solidifying its potential for rapid and noninvasive analysis of glucose in ISF toward DM screening.

The performance of bilayer SERS‐MN for glucose detection was further evaluated in mice, and the blood glucose levels also were determined by a commercial glucometer for comparison. The glucose level in ISF recorded by SERS‐MN was 3.2 mm at the fasting condition (Figure 4G). After intraperitoneal injection of glucose solution (2 g/kg), the ISF glucose level increased sharply and reached 11.5 mm at 30 min, and subsequently slowly decreased to the normal value within 2 h. The glucometer depicted a similar response to the glucose level in ISF. Next, to explore whether the bilayer SERS‐MN can be used to trace the treatment process of diabetes, a diabetic mice model was established, and ISF glucose levels was monitored before and after insulin injection. Compared with normal mice that can secrete sufficient insulin (Figure S17), the ISF glucose level of diabetic mice remained persistently high (Figure S18). After insulin treatment, ISF glucose decreased noticeably and returned to normal level within 30 min (Figure 4H). The similar trend of blood glucose determined by a glucose meter further validated the availability of bilayer SERS‐MN for monitoring glucose level in vivo. It was notable that the glucose levels in ISF was slightly lower than those in serum (Figure S19), accounting for the fact that blood glucose are exuded into ISF through the blood vessels, and thus the changes in ISF glucose levels have a certain time lag relative to blood glucose. However, compared with invasive blood glucose detection, SERS‐MN can painlessly and timely monitor the glucose level of ISF with quite well patient compliance, which is more advisable for the optimal management of diabetes.

### 2.5 Immunized SERS‐MN for Highly Sensitive Analysis of ESAT‐6/CFP‐10 Antigen

**FIGURE 5:**
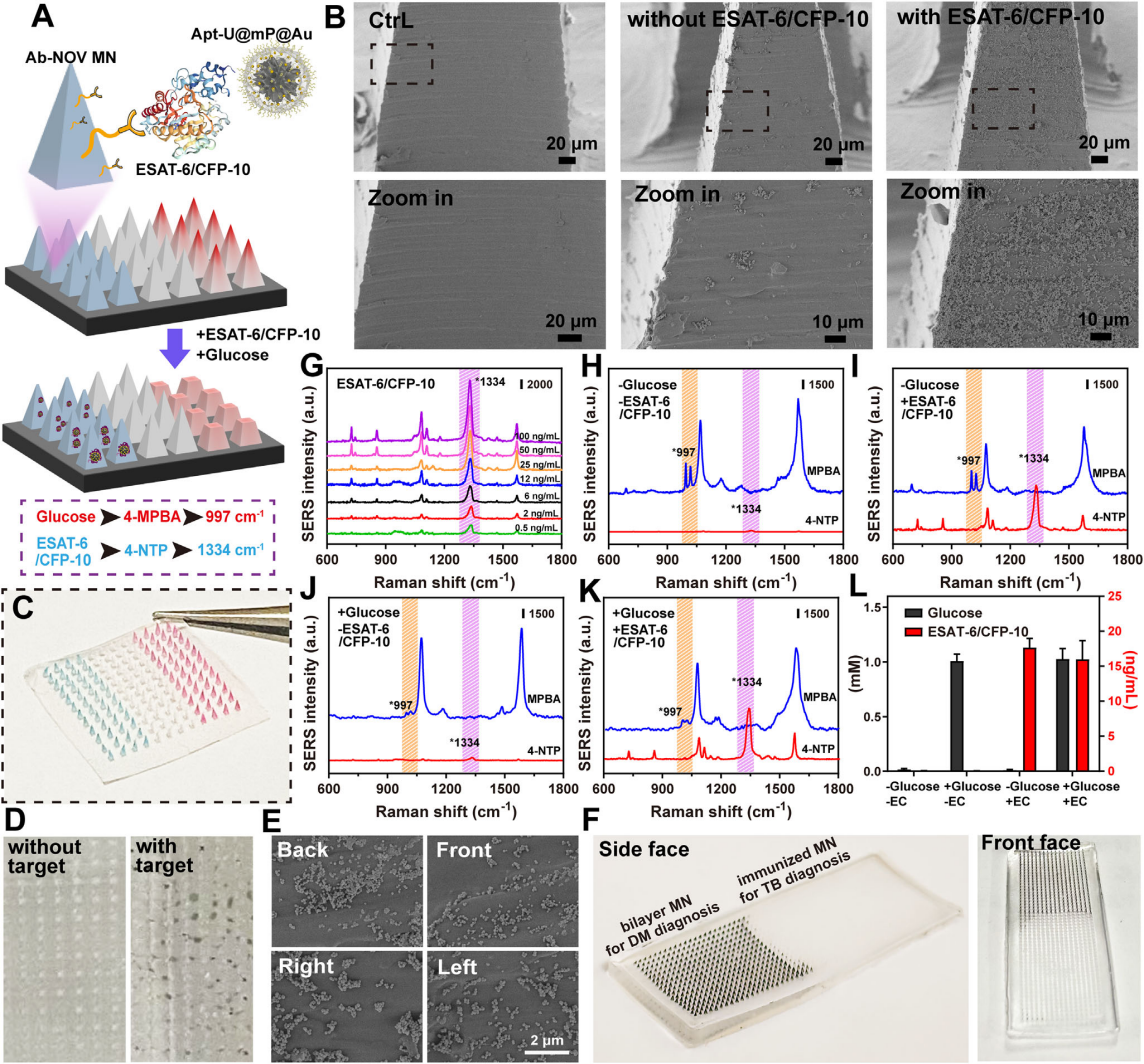
SERS‐MN for ESAT‐6/CFP‐10 detection. (A) Schematic illustration of the workflow of bilateral SERS‐MN for ESAT‐6/CFP‐10 detection in sputum and glucose detection in ISF. (B) SEM image showing the U@mP@Au aggregated on the immunized MN in the presence or absence of ESAT‐6/CFP‐10 target. (C) Optical image of the bilateral MN patch after staining. (D) The immunized MN treated with or without ESAT‐6/CFP‐10 target. (E) SEM images of U@mP@Au/ESAT‐6/CFP‐10 complexes lingering on different side of MN. (F) Different side of the bilateral MN patch. (G) SERS response of the immunized MN after incubation with various concentrations of ESAT‐6/CFP‐10. SERS spectra for bilateral SERS‐MN treated with (H) blank control, (I) glucose alone, (J) ESAT‐6/CFP‐10 alone and (K) glucose + ESAT‐6/CFP‐10. (L) Glucose and ESAT‐6/CFP‐10 levels calculation from the corresponding SERS intensity. Data are presented as mean ± SD. (*n = 3*).

*Mtb*‐specific ESAT‐6/CFP‐10 antigen complex is a protein actively secreted after early infection, and thus recognized as one of the ideal antigens for TB diagnosis [26, 27, 28]. Compared with traditional microbiological testing, such as sputum smear microscopy and culture test, direct detection of circulating ESAT‐6/CFP‐10 antigens in sputum can avoid complicated sampling and viable bacterial cells test problems. However, these antigens exist in vivo in the form of antigen complexes with low circulating concentrations, which limits their detection by standard immunoassays. Therefore, SERS technique introduced in this study provided a novel approach for sensitive and quantitative detection of the circulating ESAT‐6/CFP‐10 antigen complex in sputum. Specifically, immunized MN that was prepared by NOA polymer with mercaptoester functional group can crosslink antibodies for selectively capture of ESAT‐6/CFP‐10. ESAT‐6/CFP‐10 antigen in sputum was adsorbed by the aptamer‐functionalized U@mP@Au, and bound on the immunized MN to form MN/ESAT‐6/CFP‐10/U@mP@Au sandwich nanocomposites (Figure 5A). The U@mP@Au agglomerating on the MN surface generated a strong SERS signal under laser irradiation, allowing highly sensitive analysis of ESAT‐6/CFP‐10 antigen by SERS sensing.

The availability of this immunized SERS‐MN was tested by SEM and presented in Figure 5B. In the absence of ESAT‐6/CFP‐10, U@mP@Au cannot form an effective conjugation with MN. However, the number of U@mP@Au increased sharply on MN surface with the addition of the target protein due to the formation of sandwich nanocomposites. Microscopy further supported that a large number of black dots (U@mP@Au) were distributed on the immunized MN (Figure 5D). For SERS assessment, Raman tag (4‐NTP) was labeled on U@mP@Au with the characteristic SERS peak at 1334 cm^−1^ (Figure 5A). The SERS signal appeared when U@mP@Au was attached to the immunized MN, in agreement with that observed from SEM images. It was noted that there was no significant change in SERS intensity after aptamer modification, suggesting aptamer decoration would not affect the local electromagnetic field of U@mP@Au (Figure S20). The optimal antibody concentration modified on MN was 2 mg/mL, and the optimal aptamer concentration decorated on U@mP@Au was 10 µm (Figure S21).

The conjugation time of the target was further optimized, and the results showed in Figure S22 revealed that the test was completed within 1 h based on the high binding efficiency of U@mP@Au and ESAT‐6/CFP‐10. The SERS signal obtained from the MN surface was uniform and stable (Figure S23), which was attributed to the even distribution of U@mP@Au on different sides of MN (Figure 5E). To evaluate the stability of ESAT‐6/CFP‐10 detection, U@mP@Au and MN were stored at 4°C for 20 days, and then allowed to form the sandwich nanocomposites again, followed by SERS determination (Figure S24). The similar SERS results illustrated the good reproducibility and stability of our method. Then, the sensitivity of the immunized SERS‐MN was further studied. The SERS intensity of 4‐NTP (at 1334 cm^−1^) augmented tempestuously with the increase in ESAT‐6/CFP‐10 concentration from 0.5 to 100 ng/mL (Figure 5G). The regression equation was Y = 218.36X + 2781.9 (Y represented the Raman intensity at 1334 cm^−1^; X represented the ESAT‐6/CFP‐10 concentration), demonstrating a good linear correlation (Figure S25A). The square of correlation coefficients (R^2^) was 0.979, and the calculated LOD toward ESAT‐6/CFP‐10 was 0.06 ng/mL that determined by three times the SD of the background. The effectiveness of this SERS‐MN in the quantitative detection of ESAT‐6/CFP‐10 protein was further verified by the traditional ELISA method (Figure S25B). Compared with the limited linear range (1–25 ng/mL) and the minimum detection limit (0.62 ng/mL) of the traditional ELISA method, SERS‐MN has comparable or even significantly better performance.

To meet the requirements of POCT, different concentrations of ESAT‐6/CFP‐10 were detected by a portable Raman spectrometer (Zolix FI532, China), and results showed that the SERS signal intensity also increased with the increase of ESAT‐6/CFP‐10 concentration (Figure S26A). However, the laser of the portable instrument is unstable, resulting in poor reproducibility of multi‐batch results, with an RSD of 19.2% (Figure S26B). Therefore, we still use traditional confocal Raman microscopy instruments to ensure the accuracy and reproducibility of sample detection. To further unveil this strategy for specifically responding to ESAT‐6/CFP‐10, all the interfering proteins were tested and then mixed to interfere with the detection of ESAT‐6/CFP‐10. As expected, this immunized SERS‐MN strategy demonstrated specific recognition of ESAT‐6/CFP‐10 even under interference (Figure S27), holding the enormous potential to detect ultra‐low concentrations of ESAT‐6/CFP‐10 in complex sputum samples.

At present, many new approaches for TB diagnosis based on ESAT‐6/CFP‐10 biomarker have been developed [29, 30, 31], but less attention has been paid to the detection of TB complications, especially DM complicated with TB infection. It is well known that the presence of DM is a known risk factor for enhancing the severity of TB disease; thus, TB‐DM comorbidity has become a major health concern worldwide [32]. However, there is still an information lag in the combined detection of DM and TB disease in clinical practice, resulting in prolonged treatment time for TB and increased risk of death, recurrence, and drug resistance. Taking this into account, we proposed a bilateral SERS‐MN composed of a bilayer MN and immunized MN to detect glucose and ESAT‐6/CFP‐10 for screening TB‐DM comorbid patients (Figure 5F).

For the convenience of observation, we stained both sides of the microneedle with two kinds of dyes. From Figure 5C, the right side (red) was bilayer MN used for detecting ISF glucose, and the left side (blue) was immunized MN that detected ESAT‐6/CFP‐10 based on the U@mP@Au‐mediated immune response. It was notable that the middle insoluble side (white) was specially designed to separate the two MN models because immunized MN needs to be incubated with U@mP@Au‐labeled ESAT‐6/CFP‐10 solutions, while the bilayer MN containing U@mP@Au with swellable properties might inevitably absorb the adjacent solution, thereby affecting the detection of glucose. Moreover, considering that the bilayer MN is susceptible to interference from the liquid solution, we thus first measured glucose in ISF with the bilayer MN, and then added the sputum sample into the immunized MN to test TB infection in practical applications. This means that the bilateral MN detection was carried out in sequence to avoid mutual interference between the two sides, which is where the bilateral SERS‐MN operated smoothly.

To verify the feasibility of this integrated dual‐analyte detecting platform, bilayer MN was inserted in glucose (1 mm) pre‐incubated gelatin for glucose detection; and then, immunized MN was incubated with ESAT‐6/CFP‐10 (20 ng/mL) spiked sputum for TB detection. When the samples did not spike glucose and ESAT‐6/CFP‐10, the bilateral SERS‐MN patch showed a negligible change for the corresponding characteristic SERS peaks in either side (Figure 5H). Glucose or ESAT‐6/CFP‐10 addition alone exhibited the decrease of 4‐MPBA peak (997 cm^−1^) at the right side or the emergence of 4‐NTP peak (1334 cm^−1^) at the left side (Figure 5I,J). The bilateral SERS‐MN displayed an evident SERS change on both sides when the two targets coexist (Figure 5K), demonstrating superb capability for TB‐DM co‐diagnosis. The SERS quantified information calculated by the standard curve further corroborated a favorable correlation between the SERS value and the glucose and ESAT‐6/CFP‐10 concentration (Figure 5L), which would be helpful for further analysis of the clinical sample.

### 2.6 Bilateral SERS‐MN Available to Screen TB‐DM Comorbidity in Clinical Samples

**FIGURE 6:**
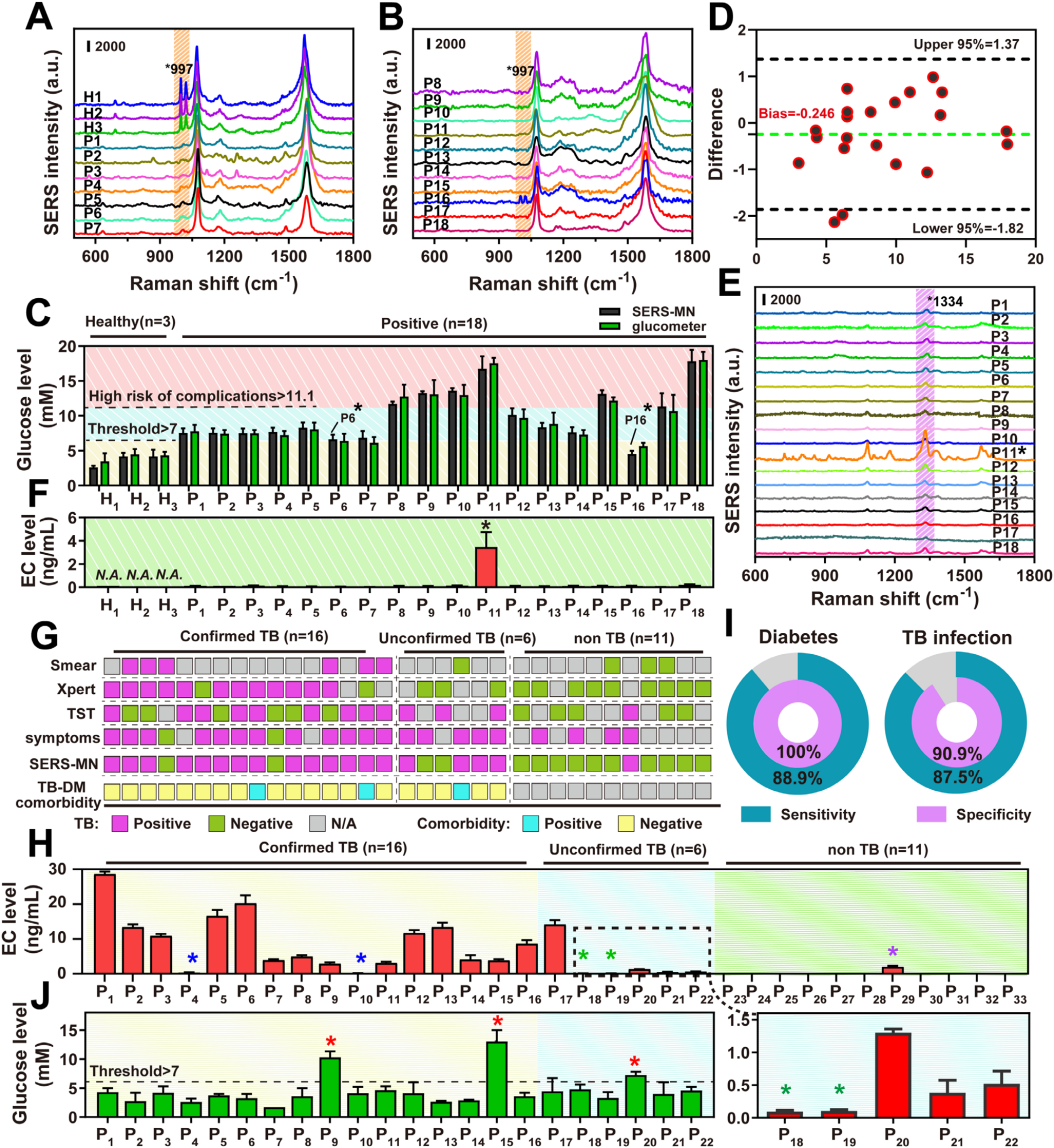
Clinical samples analysis for screening TB‐DM comorbidity. (A, B) SERS spectra of different DM participants detected by bilayer SERS‐MN. (C) Quantification of ISF glucose in different DM participants by bilayer SERS‐MN. Data are presented as mean ± SD. (*n* = 3). (D) Bland‐Altman analysis for the differences between SERS‐MN and glucometer detection. (E) SERS spectra of different DM participants detected by immunized SERS‐MN. (F) Quantification of ESAT‐6/CFP‐10 in different DM participants by immunized SERS‐MN. *N.A*. indicates not applicable. Data are presented as mean ± SD. (*n = 3*). (G) Cluster map of clinical and SERS‐MN test results for all samples. Clinical tests include smear, Xpert, TST, and clinical symptoms. DM complication was also assessed after TB analysis, where blue block and yellow block indicate the positive and negative of TB‐DM comorbidity, respectively. (H) Quantification of ESAT‐6/CFP‐10 in different TB participants by immunized SERS‐MN. Image from right bottom enlarges the ESAT‐6/CFP‐10 results of unconfirmed TB patients. Data are presented as mean ± SD. (*n* = 3). (I) Sensitivity and specificity of DM or TB detection. (J) Quantification of ISF glucose in different TB participants by bilayer SERS‐MN. Data are presented as mean ± SD. (*n* = 3).

Herein, to further assess the SERS‐MN sensing platform for DM diagnosis, an in‐depth study of 21 samples, including 3 healthy individuals (H1‐H3), and 18 positive patients (P1‐P18) were conducted to detect the glucose levels in ISF. The bilayer MN patch was inserted into the skin of patients to acquire SERS profiles. It can be seen that all healthy subjects showed the distinct fingerprint identification band at 997 cm^−1^ (Figure 6A), while the characteristic peak declined remarkably in DM patients (Figure 6B), implying the high reliability and accuracy of bilayer SERS‐MN for glucose detection. Each SERS spectra were measured three times, and the glucose value was converted from the standard curves of SERS ratio (I_1071_/I_997_). The normal plasma glucose level in healthy individuals is generally from 3.9 to 6.1 mm at fasting, while a value >7 mm will increase the risk of DM, and complications may occur when the normal value is raised to 11.1 mm.

As expected, the glucose level in ISF determined by bilayer SERS‐MN was in concordance with the results of the commercial glucometer. Bland‐Altman analysis was conducted by plotting the average concentration ratio of our SERS assay and glucometer assay. Most of the measured data were included within the 95% confidence interval, demonstrating the good conformity between two analytical methods (Figure 6D). The glucose value of DM patients was substantially upregulated compared to the healthy participants, except for two positive specimens (P6 and P16, sign in black stars) whose glucose value in ISF was below 7 mm (Figure 6C, Table S2); these two patients were deemed false‐negatives due to subsequent confirmation of positive status upon further review of the clinical records. The performance of the bilayer SERS‐MN was conducted to evaluate its diagnostic capability by using receiver operating characteristic (ROC) curves. The result showed that with regard to this cohort, the area under the curve (AUC) was calculated to be 0.946 (95% CI, 0.89–1.00), with 88.9% (16/18) sensitivity and 100% specificity (3/3), which elucidated the effectiveness of our SERS‐MN for identifying DM in clinical samples (Figure 6I, Figure S28A). Notably, compared with the poor patient compliance of traditional finger pricking blood examination, this SERS‐MN painless and bloodless monitoring of glucose in ISF provided a supplementary assessment of blood glucose levels for DM patients.

Furthermore, we noticed that among the recruited patients, six patients (P8, P9, P10, P11, P15, and P18) with high glucose levels > 11.1 mm are potentially at high risk for DM complications. Considering the risk of DM comorbidity in TB patients, *Mtb*‐derived ESAT‐6/CFP‐10 levels in the sputum of these DM patients were subsequently examined by immunized SERS‐MN to screen for potential TB‐DM comorbidity (Figure 6E). As shown in Figure 6F, all DM patients were identified as negative for TB infection according to the coded SERS signal (1334 cm^−1^ for 4‐NTP), while only P11 patient, who exhibited abnormal ESAT‐6/CFP‐10 values, indicated a relatively high risk of harboring TB disease.

To further assess the reliability of ESAT‐6/CFP‐10 levels for TB diagnosis and screening TB‐DM comorbid patients, sputum specimens with or without TB infection were collected by Dongguan Ninth People's Hospital (Dongguan Infectious Disease Hospital) and analyzed. This cohort recruited 33 samples who were retrospectively categorized into “confirmed TB” (*n* = 16) and “non TB” (*n* = 11) groups on the basis of clinical evidence, respectively; and patients only with TB suspicious symptoms, or immunological evidence of TB infection (TST test), or a positive response to treatment for TB were included in the “unconfirmed TB” (*n* = 6) groups. All of these enrolled participants underwent the ESAT‐6/CFP‐10 and glucose test to screen the TB‐DM comorbidity. The number of positive (pink blocks) and negative (green blocks) TB diagnoses identified by ESAT‐6/CFP‐10 values in SERS‐MN method, along with results of clinical trials (TST, Xpert, and smear) were listed in Figure 6G (grey blocks represent no available data). ESAT‐6/CFP‐10 values of all participants were derived based on the SERS linear relationship and are shown in Figure 6H, Table S3, where the “unconfirmed TB” patients with extremely low ESAT‐6/CFP‐10 signals were magnified beside the main image for clear observation.

In the “confirmed TB” group, all the patients were identified successfully, with the exception of P4 and P10, who had nearly undetectable ESAT‐6/CFP‐10 values (marked by blue stars) (Figure 6H). It was notable that these two patients had inconspicuous symptoms of TB disease or negative TST, whereas the later Xpert results all confirmed TB positive. Among the 11 samples confirmed as “non TB”, ten were tested as negative by the SERS‐MN assay, while only one patient (P29, marked by purple stars) with high ESAT‐6/CFP‐10 value was reassigned to “unconfirmed TB,” whose TST testing and clinical symptoms were also positive. We speculated that this case might be a latent TB infection, or possibly of incipient/nonsevere TB, which is highly deceptive in the detection of active TB infection. As no more information on Xpert and smear data was provided, the P29 patient still need further identification in the later stage. Considering P4 and P10 patients were deemed as false‐negatives, and P29 patient should be reclassified into the “unconfirmed TB” group, 87.5% of sensitivity (14 of 16 cases) and 90.9% of specificity (10 of 11 cases) was calculated by ROC curve for TB diagnosis relying on ESAT‐6/CFP‐10 levels, with 0.945 of AUC (95% CI, 0.88‐1.00) (Figure 6I, Figure S28B). The sensitivity of SERS‐MN was significantly higher than that of TST test (60%, 9 of 15 cases), and comparable to that of the Xpert (85.7%, 12 of 14 cases). Although smear testing has a 100% sensitivity, it is unreliable considering the hospital did not provide complete patient information, and it is susceptible to patients with a lower bacterial load.

In the six patients of the “unconfirmed TB” group, four were positive as measured by SERS‐MN, which coincided with positive results from the follow‐up clinical testing; two patients (P18 and P19, highlighted by green stars) have barely quantifiable ESAT‐6/CFP‐10 levels despite the TST test and clinical symptoms being positive, collected at the initial stage of the study (Figure 6H). These two cases did not show significant improvement in the subsequent anti‐TB treatment, thus they were redefined as the “non TB” group. Considering four patients (P17, P20, P21, and P22) were redefined as “confirmed TB” group, the sensitivity of SERS‐MN assay was recalculated at 81.8% (18 of 22 cases) in patients who received TB treatment (confirmed‐plus‐unconfirmed TB, who were treated at the time of baseline sample collection). Overall, SERS‐MN demonstrated remarkable diagnostic performance for TB, with the sensitivity superior to current clinical methods, including TST and Xpert.

While conducting the TB inspection, the glucose level of patients from the “confirmed TB” and “unconfirmed TB” group were also collected to further evaluate the potential of TB‐DM comorbidity. It was obvious that three TB‐positive patients (P9, P15, and P20, tagged by red stars) have a high likelihood of DM disease, which is highly predictive of the TB‐DM comorbidity (Figure 6J). Notably, the probability of screening for DM comorbidity from TB patients is slightly higher than that from DM patients for TB infection (Figure 6F), which is reasonable because the patients have to undergo a routine inspection after diagnosis with TB infection, making it easier to find out the elevated blood glucose levels. Besides, we found that TB‐DM comorbidity is also accompanied by other complex diseases, such as Syphilis, Klebsiella pneumoniae, and Candida albicans infections, as TB infection is caused by weakened immunity and is more likely to be complicated with other diseases (Table S3).

TB comorbidity is actually not uncommon in clinical practice. However, the department of respiratory infectious diseases paid more attention to the pulmonary TB infection and often neglected the complications of DM, lacking an effective combined diagnostic strategy in clinical practice. Therefore, unlike the current studies that only focus on the TB diagnosis, these cases emphasized the advantages of SERS‐MN test in the co‐diagnosis of TB‐DM, providing a novel assay for the TB‐DM comorbidity.

## 3 Conclusion

In summary, we herein developed a core‐satellite structure of U@mP@Au with robust, stable, and reproducible SERS signals, and conjugated them with a bilateral MN patch for minimally invasive detection of glucose in ISF and ESAT‐6/CFP‐10 biomarker in sputum toward cooperative diagnosis of TB‐DM comorbidity. Unlike the current technologies that can only test TB or DM separately, this SERS‐MN array implemented two kinds of detection strategies on two sides of the patch, respectively, and detected TB and DM by recording the corresponding SERS response. More importantly, this bilateral SERS‐MN patch can be customized to assemble other probes or covalently linked with antibodies to capture other biomarkers, further expanding the application range of liquid biopsy in disease diagnosis. However, although we have conducted application exploration on pH detection, the guiding effect of pH on TB‐DM comorbidity is extraordinarily weak, and thus, no in‐depth clinical connection has been constructed in our study. Additionally, a viscous sputum sample must be diluted or lysed in advance during the detection process, especially for samples with severe TB infection, which undoubtedly affects the analytical concentration of the biomarker and also increases the complexity of the operation. In the following research, we will integrate the proposed methodology with a miniaturized POCT device to automate the pretreatment of clinical samples, signal detection, and analysis.

## 4 Materials and Methods

### 4.1 Synthesis and Functionalization of U@mP@Au

The U@mP@Au was prepared in three consecutive stages as described in Scheme 1A, including urchin‐shaped Au/Ag alloy (UAA), core–shell UAA@mPDA NPs (U@mP), and Au seed doped U@mP (U@mP@Au). To begin, UAA NPs were fabricated based on a seed‐mediated growth and fast reduction [33]. Briefly, 9 mg Ag NO_3_ solutions were heated to boil under rapid stirring, followed by quick injection of 1% trisodium citrate, and maintained at this temperature for 2 min. The color change from colorless to green‐yellow demonstrated the formation of Ag NPs. Afterward, the resulting Ag NPs were added drop by drop to an aqueous solution containing 4.8 mL HAuCl_4_ (10 mm) and 4.8 mL L‐Dopa (10 mm), and the solution was stirred for 10 min. The fabricated UAA NPs were collected by centrifugation and purified by repeated washing with formic acid, ammonia, and H_2_O.

For synthesis of U@mP, a PDA shell was self‐polymerized on the surface of UAA NPs, followed by etching to form the mesoporous structure [34]. In detail, 50 mg of F127 and 75 mg of dopamine hydrochloride (DA) were dispersed in a mixture of 10 mL of H_2_O/ethanol. 0.08 mL 1, 3, 5‐TMB solution was then mixed into the above solution under ultrasonication. Next, the resultant UAA NPs were added into the mixture under stirring, followed by adding 0.375 mL of ammonia rapidly to trigger DA polymerization, and the solution turned pale brown immediately. The solution was continuously stirred at room temperature for 2 h, and the U@mP was collected by centrifugation, washed with ethanol and acetone.

For the synthesis of U@mP@Au, 0.1 mL of U@mP was added to 60 µL aqueous solutions containing HAuCl_4_ (25 mm), and then 60 µL of 10 mm NaBH_4_ was injected into the reaction mixture, and stirred for 15 min. The content of Au NPs embedded into the mPDA shell was constructed by changing the volume of HAuCl_4_ (5 µL, 10 ΜL, 20 µL, 40 µL, 60 µL, 80 µL). For the preparation of Raman reporter‐functionalized U@mP@Au, 1 mL of U@mP@Au was incubated with 20 µL of different Raman labels (4‐NTP, 4‐MPBA, 4‐MBA) for 4 h. The mixture was centrifuged to remove free Raman label molecules, redispersed with pure H_2_O, and stored at 4°C for further use. To selectively capture ESAT‐6/CFP‐10 antigen, U@mP@Au was linked with SH‐Apt (50 µL, 10 µm) through the Au─S bond, and incubated for 4 h with continuous shaking as in previous studies.

### 4.2 Synthesis of MN

For the synthesis of a swellable MN array for pH detection, we selected the MeHA that enabled rapidly absorbing solutions by the modification of methacrylate on HA [35]. Specifically, MeHA (50 mg/mL) and photoinitiator (LAP, 0.05%) were dissolved in PBS, and then mixed with U@mP@Au (50 µL). The mixture was cast into a PDMS mold, centrifuged at 4000 rpm for 5 min, and then exposed to UV light (405 nm). After that, 150 µL of the PVP solution was added to produce a robust supporting substrate by centrifugation. After naturally drying overnight, the MeHA/PVA patch was carefully separated from the PDMS mold and then kept in a vacuum oven before use.

For the synthesis of a bilayer MN array for glucose detection, MNs were made of the upper soluble HA and the lower swellable MeHA, where HA dissolved rapidly and was absorbed by MeHA when in contact with ISF. GOx loaded into HA can catalyze the ISF glucose to H_2_O_2_, while U@mP@Au concealed in the MeHA was used to monitor this catalytic process by SERS sensing. First, a 100 mg/mL HA solution was mixed with GOx solution (6 mg/mL), then poured into the PDMS mold. After that, the PDMS mold was centrifuged at 2500 rpm at 4°C for 5 min, followed by drying for 12 h to form the tip structure. Next, the MeHA solution (50 mg/mL) with photoinitiator (LAP, 0.05%) was mixed with U@mP@Au, and loaded in the microneedle cavities by centrifugation (4000 rpm, 4°C for 5 min). After drying, 150 µL PVA was added to the mold to form a robust backing. The bilayer patch was carefully peeled from the PDMS mold and then kept in a vacuum oven before use.

For the synthesis of an immunized MN array for ESAT‐6/CFP‐10 detection, we selected the NOA polymer that was easily conjugated with ESAT‐6/CFP‐10 Ab due to the mercaptoester functional group. Specifically, NOA 65 prepolymer solution (∼1.5 g/sample) was cast into the PDMS mold, centrifuged at 4000 rpm at 4°C for 5 min to force NOA into the needle voids and remove air bubbles. After that, the mold was placed in UV light exposure (365 nm) for 1 min to crosslink the NOA prepolymer, and NOA‐MNs patch was carefully removed from the flexible PDMS mold. The MN array was washed with ethanol, dried in air, and then immersed in Ab aqueous solution (5 µL, 2 mg/mL) overnight at 4°C. The immunized MN array was cleaned with PBS solution containing 0.05% (v/v) Tween‐20 three times to remove impurities, and cut into the optimal shape. To prepare the bilateral MN patch, the two solutions were cast in sequence, and the other half was covered with tape to prevent the solution from filling into the other side.

### 4.3 Ex Vivo Assay of SERS‐MN

For the detection of pH, swellable MN made in MeHA + U@mP@Au was pressed into agarose gel that was pre‐incubated in PBS solutions with pH levels ranging from 3 to 8. Then, MN patch was gently removed from the gel model. MN patch became swollen, and the pH‐sensitive molecule 4‐MBA functionalized U@mP@Au responded to the pH change by SERS sensing.

For the detection of glucose, bilayer MN made of upper HA + GOx and lower MeHA + U@mP@Au was pressed into agarose gel that was pre‐incubated with glucose in different concentrations from 0.2 to 20 mm. The upper HA dissolved and released GOx quickly, which further reacted with glucose in the gel to generate H_2_O_2_. After the solution was absorbed by the MeHA at the lower layer, H_2_O_2_‐sensitive molecule 4‐MPBA functionalized U@mP@Au detected the glucose level according to the H_2_O_2_ change.

For the detection of ESAT‐6/CFP‐10, 4‐NTP labeled Apt‐U@mP@Au was incubated with different concentrations of ESAT‐6/CFP‐10 antigen complexes from 0.5 to 100 ng/mL, then dropped into the antibody‐modified MN patch. After that, the MN patch was washed with PBS, and the SERS signal of 4‐NTP was acquired on the needle.

To examine the specificity and anti‐interference of the assay, diverse biologically relevant interferents, such as NaCl, KCl, CaCl_2_, and MgCl_2,_ were added to disturb glucose detection. To determine the stability, the above U@mP@Au were stored in the dark at 4°C for different days and then subjected to SERS‐MN detection. To investigate the repeatability, at least 10 different points were taken from each batch, and the average value was presented as the final result. The limit of detection (LOD) was defined as the ratio of the standard deviation of the blank to the slope of the linear equation of the calibration plot (LOD = 3 SD/slope, *n* = 3). All the SERS assays were conducted at a 633 nm laser, with 50 × telephoto objective at a power density of 16.0 mW/µm^2^, acquisition time is 2 s [36]. The effectiveness of SERS‐MN was verified by using ELISA kits. The ESAT‐6/CFP‐10 spiked sputum and HRP‐labeled antibody were added in sequence into the microplate well that was pre‐coated with ESAT‐6/CFP‐10 capture antibody. After incubation for 2h, the TMB substrate was converted into blue by the catalysis of peroxidase and then into the final yellow under the action of acid. The absorbance was measured at a wavelength of 450 nm.

### 4.4 In Vivo Assay of SERS‐MN in Real Samples

To detect the level of glucose in a mouse model, C57BL/6 mice were placed in an environment controlled by climate and light. In the experiment, the mice were intraperitoneally injected with glucose solution (2 g/kg in 200 µL PBS). After 30 min, the bilayer MN array was gently inserted onto the dorsal skin of the mouse and then removed for SERS analysis. The SERS signal was acquired on a single needle tip of the swelling MN array. To trace the treatment process of diabetes, diabetic model mice were established by feeding with high‐fat and high‐sugar diets, and intraperitoneally injecting 30 mg/kg of streptozotocin (STZ). The insulin (2 U/kg in 200 µL PBS) was injected subsequently, and the glucose level was detected by bilayer MN. The blood glucose levels of mice were also measured using a glucometer (Sinocare, China) for comparison.

To evaluate the clinical applicability of SERS‐MN for TB‐DM diagnosis, diabetic positive and negative samples (*n* = 21) were recruited from the Second Affiliated Hospital of Jinan University, and the confirmed/unconfirmed/non‐TB infected patients (*n* = 33) were received by Dongguan Ninth People's Hospital (Dongguan Infectious Disease Hospital). Patients with or without DM who show symptoms suggestive of TB are eligible. Given that the viscosity significantly affected the measurement of the sample, the sputum samples were diluted and lysed by the digestant‐decontaminant solution (containing 25 mL of 4% NaOH, 25 mL of 2.9% sodium citrate, and 0.25 g of N‐acetyl‐L‐cysteine (NALC)) at room temperature for 15 min with gentle shaking, followed by the analysis.

For the diagnosis of DM, the glucose level in ISF was monitored by bilayer SERS‐MN, and all the obtained results were compared with those measured by a glucometer. For the screening of TB, the participants were classified as “confirmed TB”, “unconfirmed TB”, or “non‐TB” through the TST test, smear microscopic examination, and PCR‐based Xpert assay. In general, the diagnosis of TB is based on a positive result of Mtb microbial culture or Xpert. However, a patient with only symptoms/signs related to TB, an abnormal TB chest X‐ray, positive TST, or a positive response to treatment for TB was considered a “unconfirmed TB” patient, and further observation is still required. All sputum samples were processed in biosafety cabinets in biosafety level 2 (BSL‐2) laboratories. The SERS signals obtained from MN were converted into the concentration of glucose or ESAT‐6/CFP‐10 according to the standard curves.

### 4.5 Ethical Statement

Informed consent was obtained from all participants prior to the commencement of any human experiments. All the experiments were conducted in accordance with clinical guidelines and approved by the local bioethics committee (YJYS202407001). All animal experiments were approved by the institutional animal care and use committee of Guangdong Medical University (GDY2602416).

### 4.6 Statistical Analysis

Data shown in figures were expressed as mean ± standard deviation (SD) from three independent replicate experiments (*n* = 3). For normally distributed data sets with equal variances, One‐way analysis of variance (ANOVA) using Tukey's post hoc test, was used for multiple group comparisons. In all cases, a significance threshold of *p < 0.05* was considered statistically significant (^*^p < 0.05, ^**^p < 0.01, ^***^p < 0.001). All statistical analyses were performed using SDSS software. ROC curves were used to determine the sensitivity and specificity of SERS‐MN, including 95% CI (confidence interval), area under the curve (AUC), and significance *p*‐value (*pROC*). Graphical representations were performed with GraphPad Prism 10.2.2 software.

## Author Contributions

X.H., L.C., J.Z., and H.Z. conceived and designed the experiments. L.C., J.Y., J.X., S.C., and H.C. performed the experiments. L.C., J.Y., Y.C., J.P., J.Z., P.S., and X.H. analyzed the data. X.H. and H.Z. wrote the manuscript. All authors have given approval to the final version of the manuscript.

## Conflicts of Interest

The authors declare no conflicts of interest.

## Supporting information




**Supporting File**: advs75722‐sup‐0001‐SuppMat.docx.

## Data Availability

The data that support the findings of this study are available from the corresponding author upon reasonable request.
