# Supplementary material for: Bilateral SERS‐Microneedle Patch for Co‐Diagnosis of Diabetes Mellitus and Tuberculosis Comorbidity
Source: Adv Sci (Weinh). 2026 May 25:e75722. Online ahead of print. doi: 10.1002/advs.75722 (PMC13335952; doi:10.1002/advs.75722)
Supplement: Supplementary file 1 — Supporting File: advs75722‐sup‐0001‐SuppMat.docx. [file ADVS-9999-e75722-s001.docx]

*Supplementary Materials* for

**Bilateral SERS-microneedle patch for co-diagnosis of diabetes mellitus and tuberculosis comorbidity**

**Authors:** Xueqin Huang ^1, 2, †^, Lingzhi Chen ^2,†^, Jing Xu ^2^ , Jiaqi Yu ^1^, Shanze Chen ^2^, Huaihong Cai ^3^, Yanguang Cong^1^, Pinghua Sun ^2,4^, Jiang Pi ^1^, Lang Rao^5^, Jierong Chen^6^*, Junxia Zheng^7^*, and Haibo Zhou ^1,2,4^*

**Affiliations:**

^1^ The First Dongguan Affiliated Hospital, Guangdong Provincial Key Laboratory of Medical Immunology and Molecular Diagnostics, School of Medical Technology, Guangdong Medical University, Dongguan, China.

^2^ State Key Laboratory of Bioactive Molecules and Druggability Assessment, Guangdong Basic Research Center of Excellence for Natural Bioactive Molecules and Discovery of Innovative Drugs, The Fifth Affiliated Hospital, College of Pharmacy, Jinan University, Guangzhou, China.

^3^ College of Chemistry and Materials Science, Jinan University, Guangzhou, China

^4^ Institute for Safflower Industry Research, Key Laboratory of Xinjiang Phytomedicine Resource and Utilization, Ministry of Education, School of Pharmacy, Shihezi University, Shihezi, China.

^5^ Institute of Chemical Biology, Shenzhen Bay Laboratory, Shenzhen, China

^6^ Department of clinical laboratory, Guangdong Provincial People’s Hospital (Guangdong Academy of Medical Sciences), Southern Medical University, Guangzhou, Guangdong, China

^7^ School of Biomedical and Pharmaceutical Sciences, Guangdong University of Technology, Guangzhou, China.

^†^ These authors contributed equally to this work.

* Corresponding e-mail: chenjierong@gdph.org.cn (J.C); junxiazheng@gdut.edu.cn (J.Z.); haibo.zhou@jnu.edu.cn (H.Z.).

**This PDF file includes:**

Materials and Reagents

Characterization and measurements

Supplementary Figure S1 to S28

Supplementary Tables S1 to S3

**Supplementary** **Materials and Reagents**

Hydrogen tetrachloroaurate (HAuCl_4_⋅3H_2_O), silver nitrate (AgNO_3_), ascorbic acid (AA), hyaluronic acid (HA, Mw 200-400 kDa), 4-mercaptophenylboronic acid (4-MPBA), 4-mercaptobenzoic acid (4-MBA), 4-nitrothiophenol (4-NTP), trisodium citrate, L-Dopa, dopamine hydrochloride, glucose and phosphate-buffered saline (PBS) were obtained from Macklin (Shanghai, China). CaCl_2_, MgCl_2_, KCl and NaCl were purchased from Alfa Aesar Chemical Co., Ltd (Shanghai, China). ESAT-6/CFP-10 antigen complex was purchased from gene-optimal Co. Ltd (Shanghai, China). ESAT-6/CFP-10 aptamer (5’-SH-GCCTGTTGTGAGCCTCCTAACCCCATCTTATAC GTATATGGACTCATCTCGACCCCCGATAGGCTTGGTACATGCTTATTCTTGTCTCCC-3’) was synthesized by Sangon Biotechnology Co. Ltd (Shanghai, China). Methacrylate hyaluronic acid (MeHA) was purchased at EFL-Tech Co., Ltd (Suzhou, China). Norland Optical Adhesive (NOA) was purchased at Norland Product Inc (Jamesburg, USA). The clinical samples from health human or DM patients were supplied by the Second Affiliated Hospital of Jinan University (Shenzhen, China). The sputum samples from confirmed or unconfirmed TB patients were collected by the Dongguan Ninth People’s Hospital (Dongguan, China).

**Supplementary** **characterization and measurements**

The morphology and element composition of U@mP@Au were characterized by field-emission transmission electron microscopy (TEM) (JEM-2100F, Japan) equipped with EDX, and XPS analysis (PHI 5000 Versa Probe, USA). Nitrogen (N_2_) adsorption-desorption isotherms of U@mP@Au was tested with an ASAP2010 analyzer (Micromeritics, USA). The morphology and size of MN patches were measured by scanning electron microscopy (SEM) (JSM-7500F, Japan), or optical stereomicroscope (SZX16, Japan). The mechanical properties of MN patches were determined by using an electronic testing machine (UTM4103, China). To perform the insertion test, the MN was vertically inserted into a piece of isolated porcine skin by thumb for 5 min. After removing the MNs, the penetrated porcine skin was embedded in paraffin blocks, sectioned, and stained for hematoxylin-eosin (H&E) examination. To confirm the bilateral MN, the MN patch stained with malachite green and R6G were observed using an optical microscope. The soluble and swelling effect was observed at each determined time after the PBS solution addition. The biosafety and biocompatibility of MN was evaluated by immersing MN into the HUVEC cell (1 × 10^6^). The cell viability after treatment was measured using the CCK-8 assay.

**Supplementary** **calculation of enhancement factor**

The enhancement factor (EF) was calculated based on this equation

$$EF=\frac{I_{SERS}\times N_{bulk}}{I_{bulk}\times N_{SERS}}$$

where *I_bulk_* and *I_SERS_* represent the bulk Raman spectral intensity and SERS intensity of the analyte in solution, respectively. *N_bulk_* and *N_SERS_* respectively represent the number of molecules in the spots excited by the laser spots in bulk Raman spectra and SERS.

$$N_{SERS}=N_{A}\times CV\frac{S_{Laser}}{S_{sub}}$$

*N_A_* is Avogadro constant; C and V are the molar concentration and volume, respectively; *S_Laser_* and *S_Sub_* indicate the size of the laser spot and substrate, respectively. Therefore, 4-MPBA with V_SERS_ volume and C_SERS_ concentration is dispersed on a clean Si substrate.

$$N_{bulk}=N_{A}\times P_{v}S_{Laser}$$

*Pv* [mol/μm^3^] represents the volume density of 4-MPBA powder on a glass slide. During this calculation process, mass density of 4-MPBA powder is 1.27 g/cm^3^, while molecular weight of 4-MPBA is 153.1 g/mol, thus it can be calculated as *Pv* [mol/μm^3^] = (1.27/153.1) × 10^-12^ = 8.30 ×10^-15^ mol/μm^3^.

$$EF=\frac{I_{SERS}\times P_{v}S_{sub}}{I_{bulk}\times CV}$$

In this experiment, 20 μL of 4-MPBA (1×10^-9^ M) was incubated with U@mP@Au substrate, and dried in the air to form a circle with a diameter of 4728 μm. The Raman signal of 4-MPBA was analyzed, where *I_SERS_* (10428) was significantly stronger than that of *I_bulk_* (672) at the characteristic peak of 1573 cm^-1^. Therefore, EF is calculated as: EF = (10428 counts×8.30×10^-15^ mol/μm^3^×(4728 μm)^2^×3.14)/(672 counts×1.0×10^-9^ M×20 μL×10^-6^) =4.52×10^8^.

**Supplementary Figures and Tables**


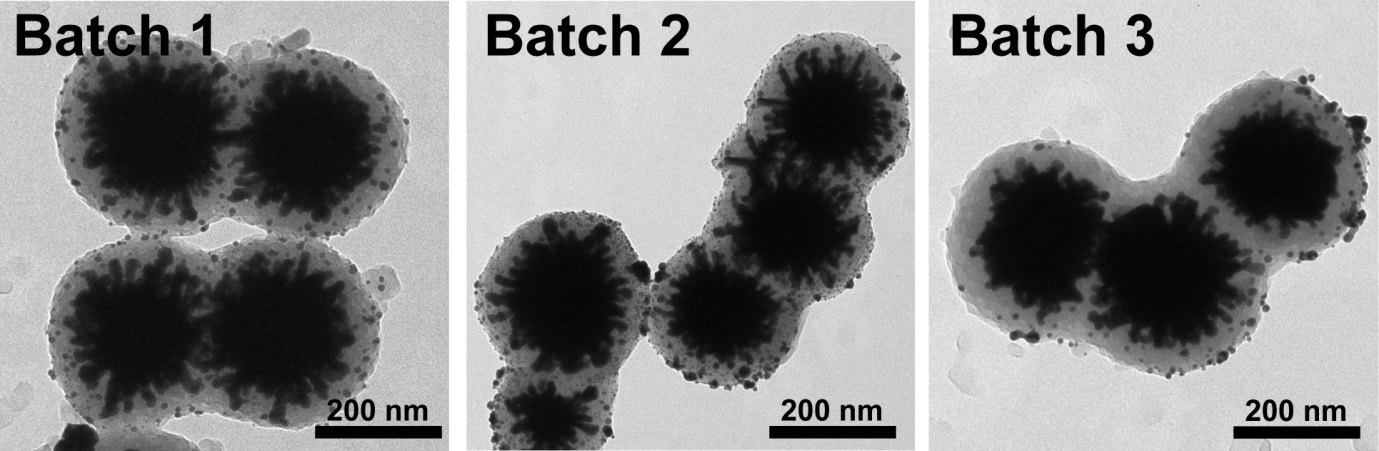


**Supplementary Figure 1.** TEM images of U@mP@Au in different batches.


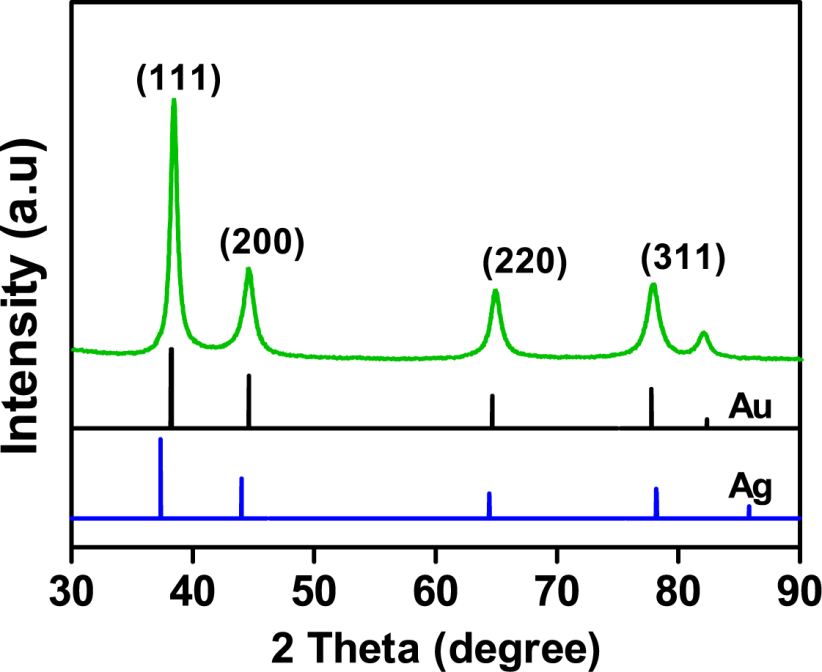


**Supplementary Figure 2.** XRD pattern of U@mP@Au.


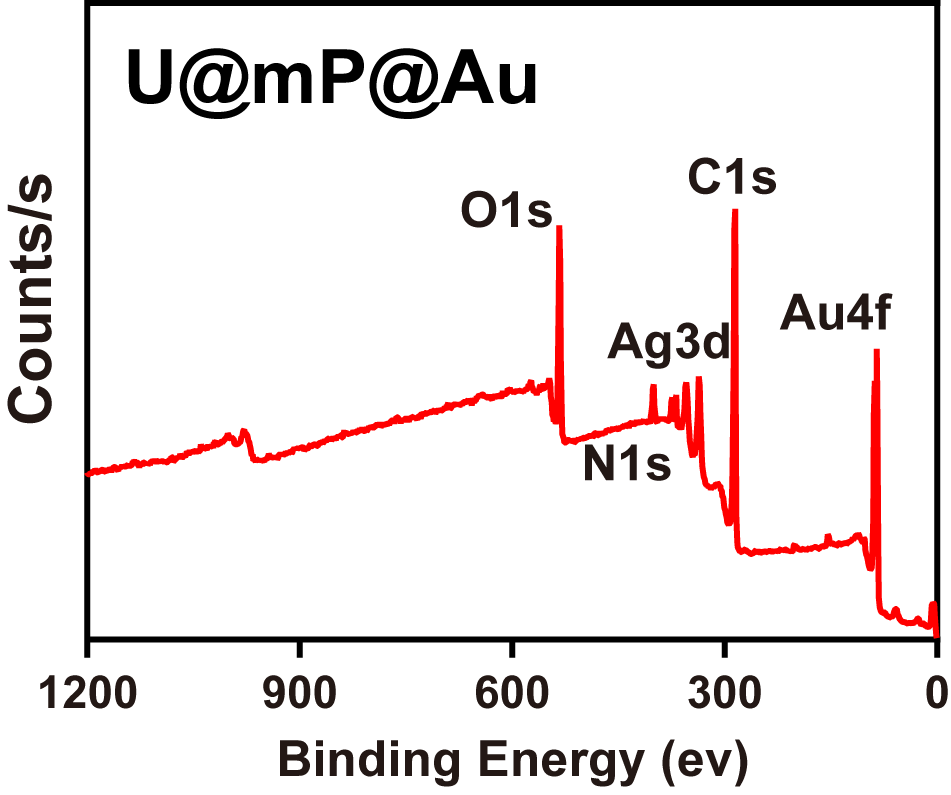


**Supplementary Figure 3.** XPS survey scan of U@mP@Au.


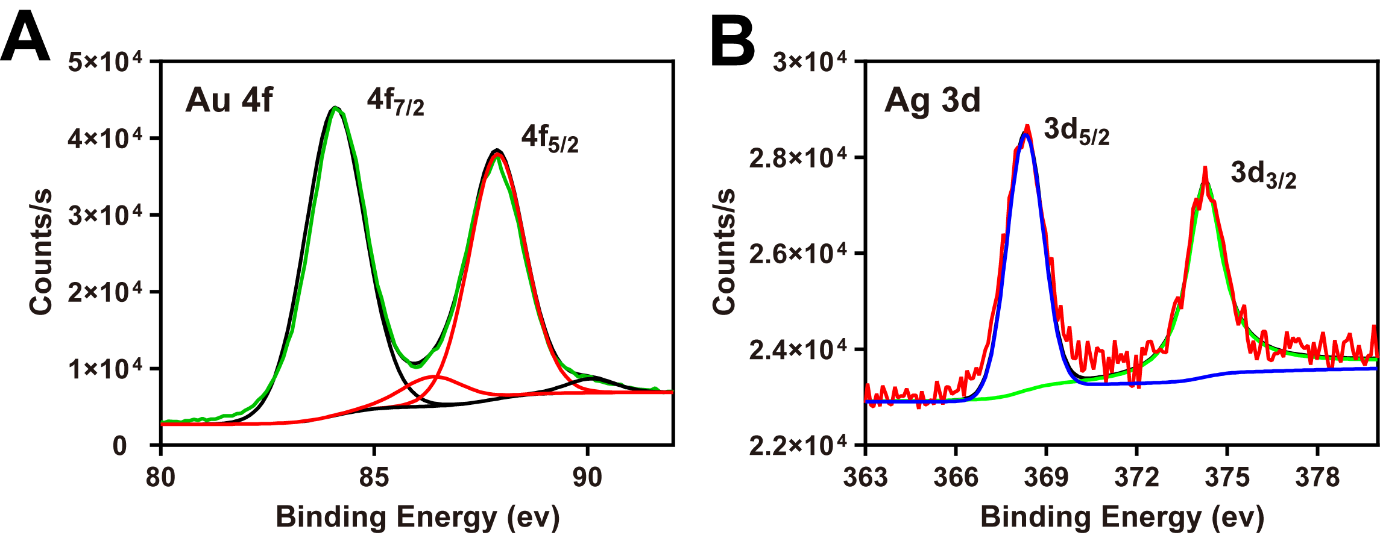


**Supplementary Figure 4.** High-resolution (A) Au 4f and (B) Ag 3d XPS spectra of U@mP@Au.


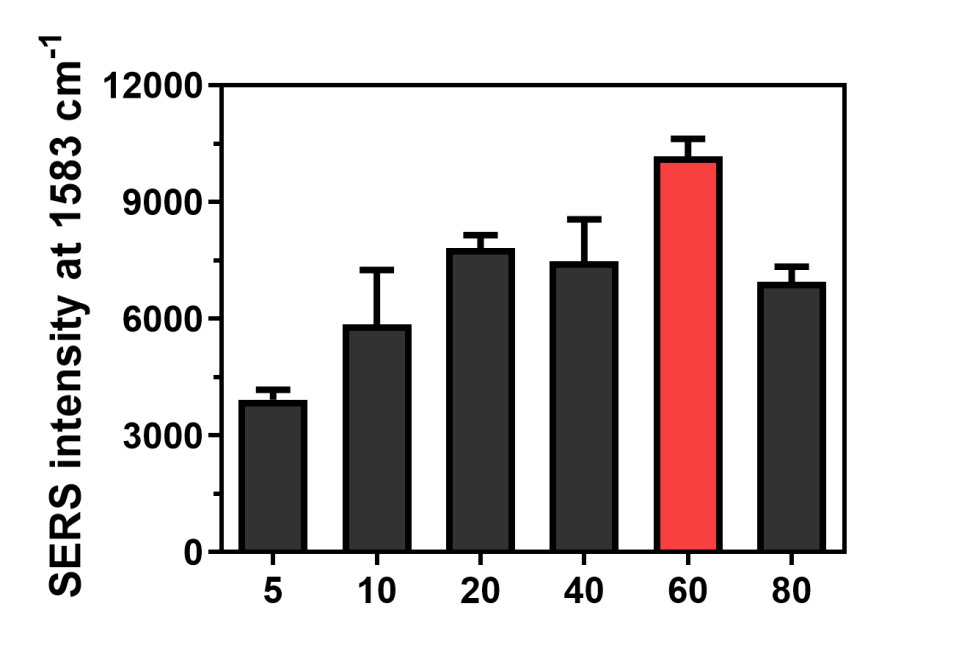


**Supplementary Figure 5.** SERE intensities at 1583 cm^-1^ of U@mP@Au synthesized with different amount of HAuCl_4_ (5, 10, 20, 40, 60, 80μL). All data are presented as mean ± S.D. (*n* = 3).


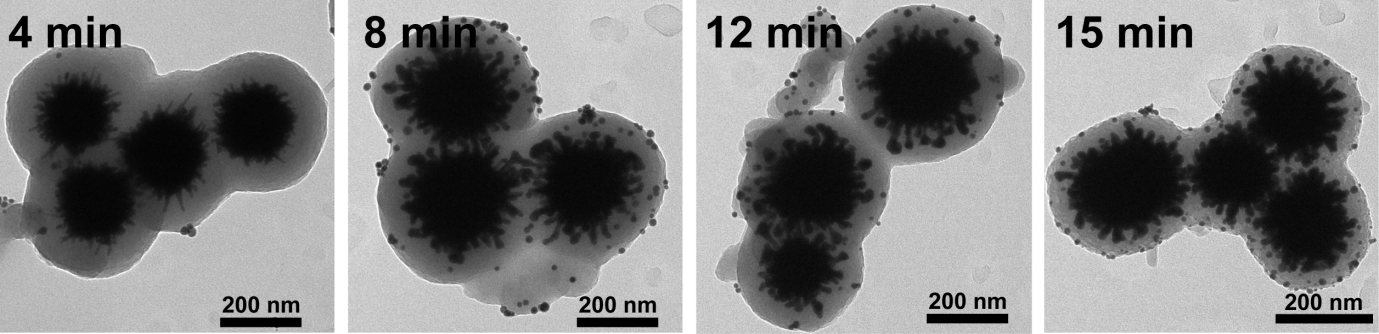


**Supplementary Figure 6.** TEM images showing the growth of Au seed on U@mP@Au at different times.


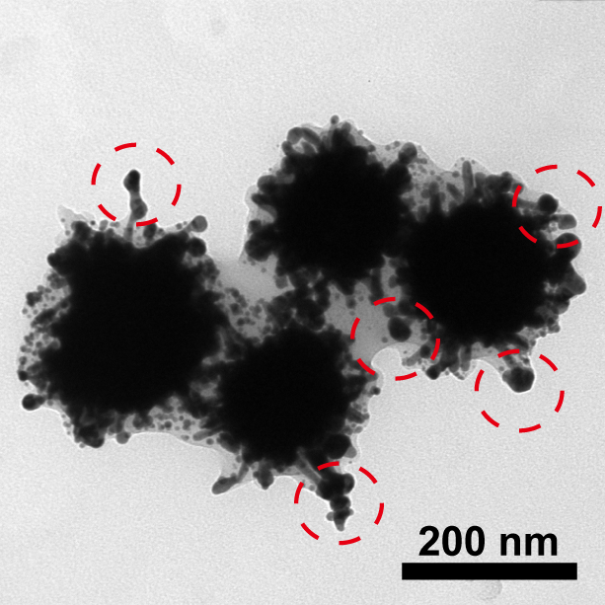


**Supplementary Figure 7.** TEM images showing the Au seed preferentially deposited at the spire of U@mP@Au.


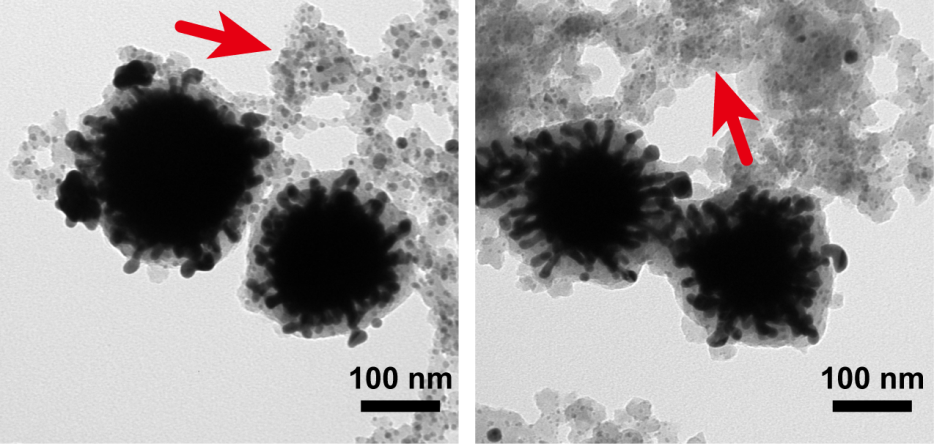


**Supplementary Figure 8.** TEM images showing mPDA self-polymerizes to form clumping layer when the content of UAA in the system was insufficient.

**
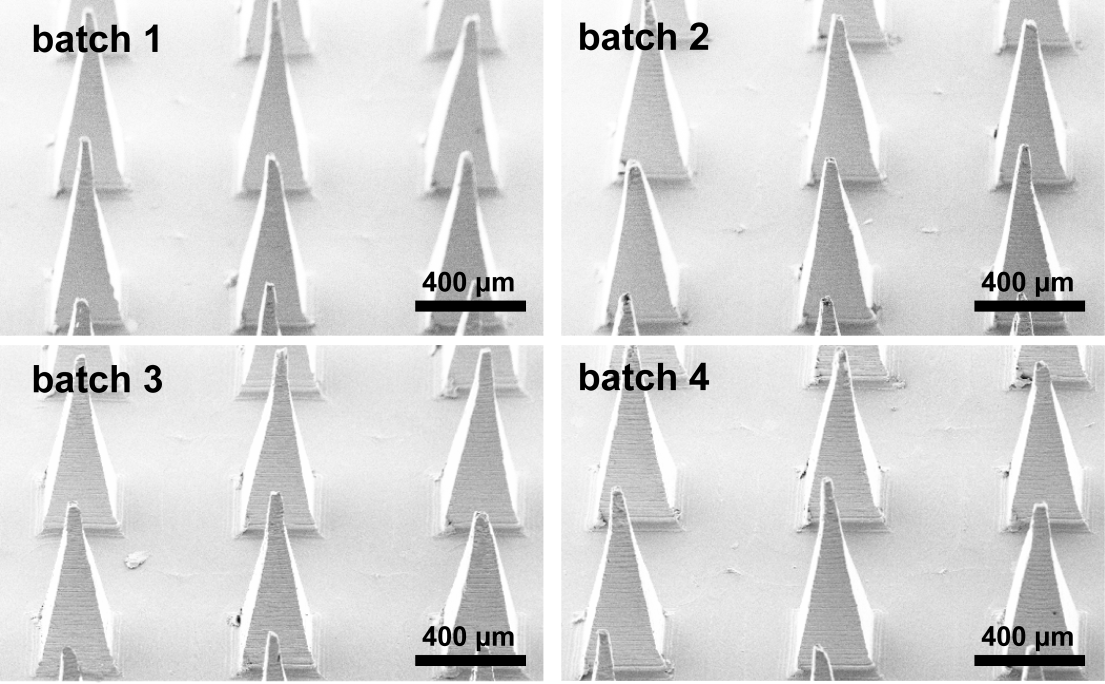
**

**Supplementary Figure 9.** SEM images of MN in different batches.


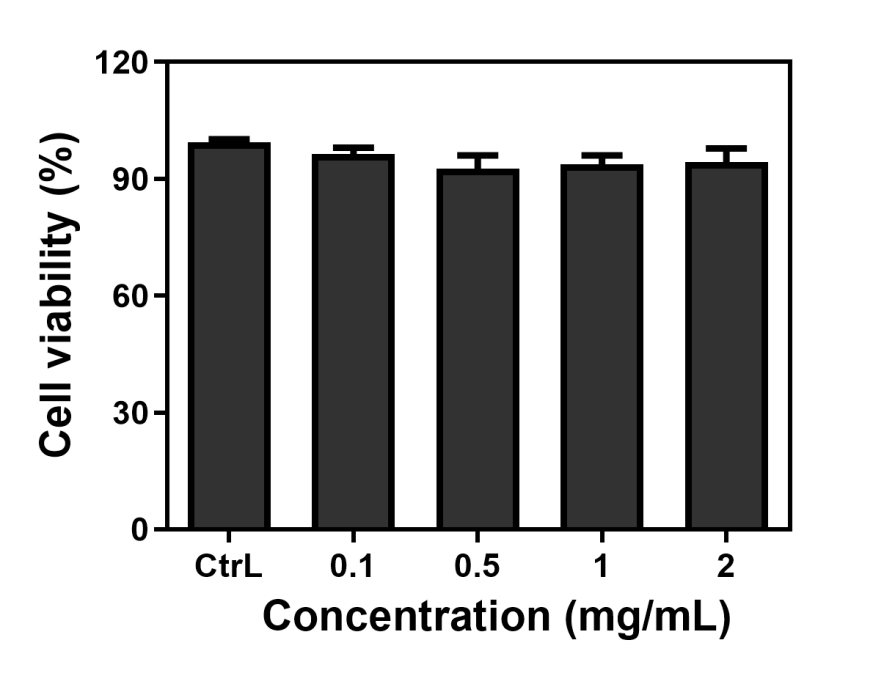


**Supplementary Figure 10.** Viability of HUVEC cells incubated with MN for different time. All data are presented as mean ± S.D. (*n* = 3).


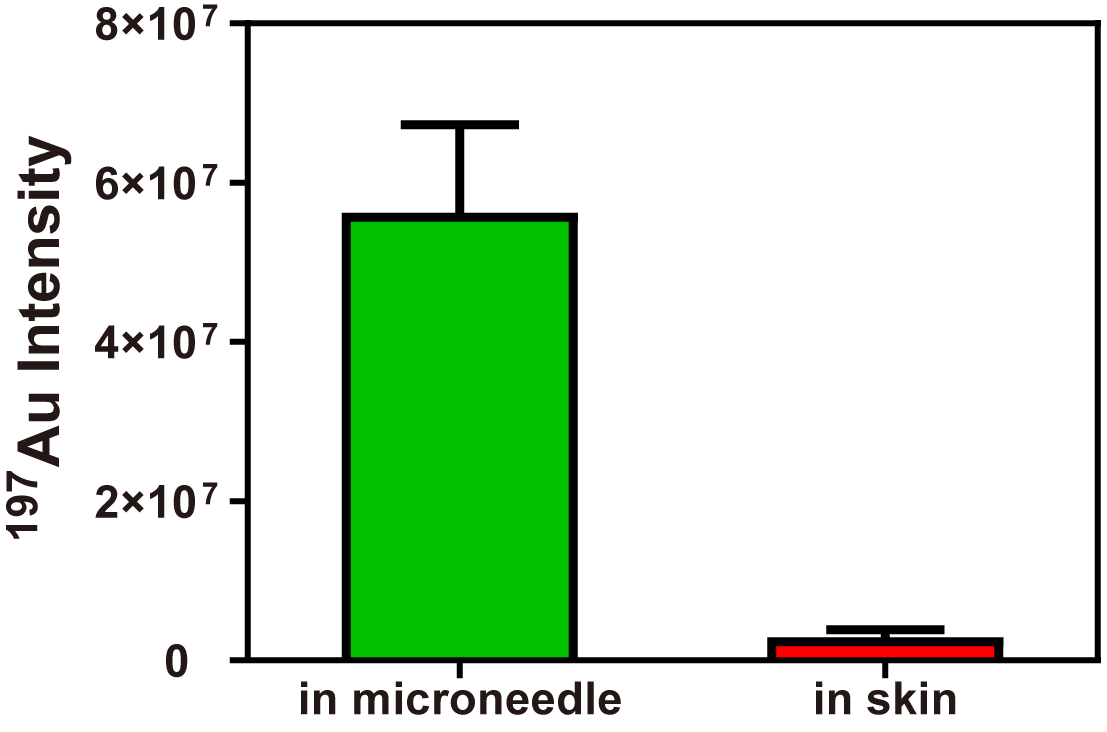


**Supplementary Figure 11.** The Au^+^ concentration remained on the porcine skin recording by ICP-MS after MN insertion. All data are presented as mean ± S.D. (*n* = 3).


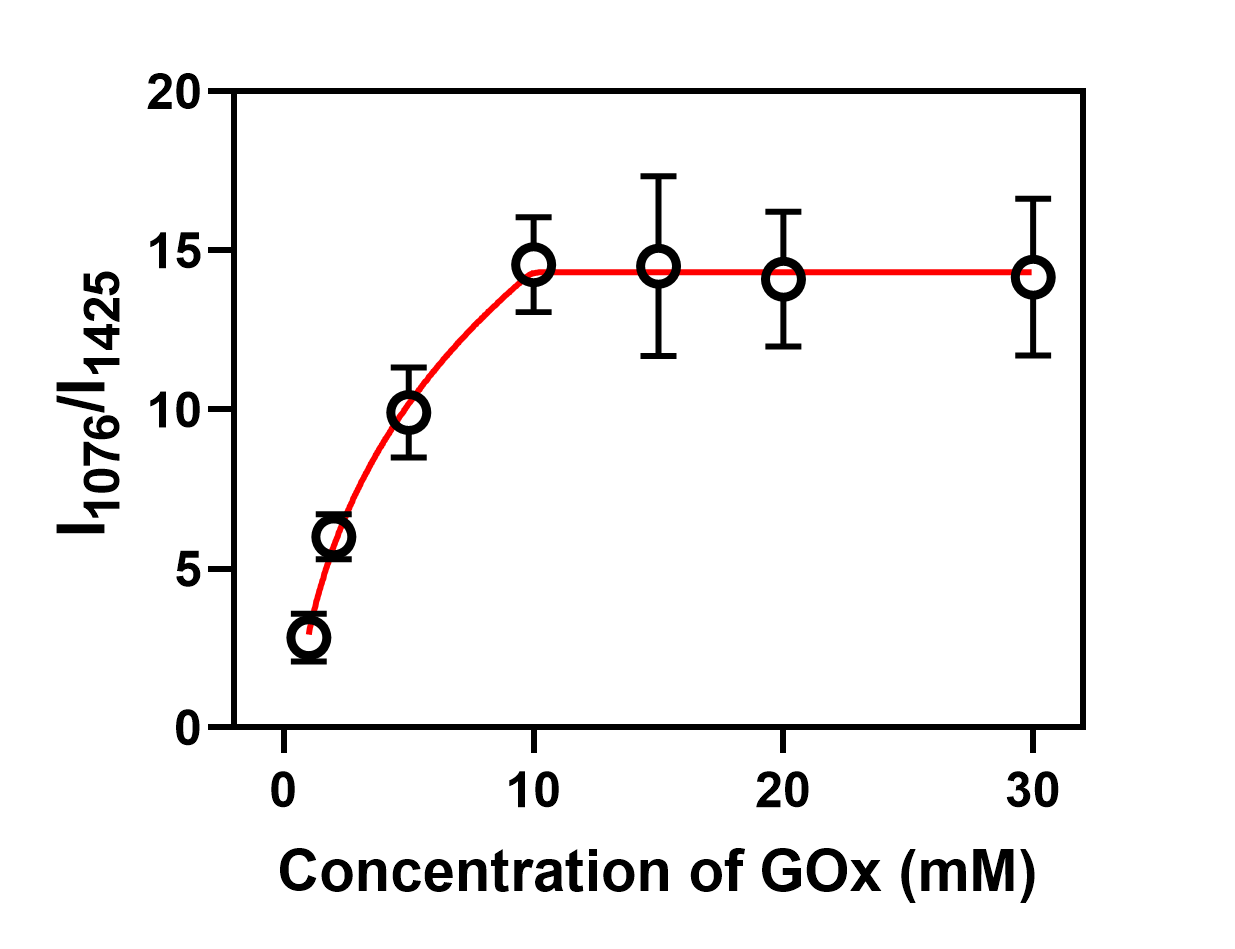


**Supplementary Figure 12.** Optimization of the concentration of GOx for catalyzing with glucose. All data are presented as mean ± S.D. (*n* = 3).


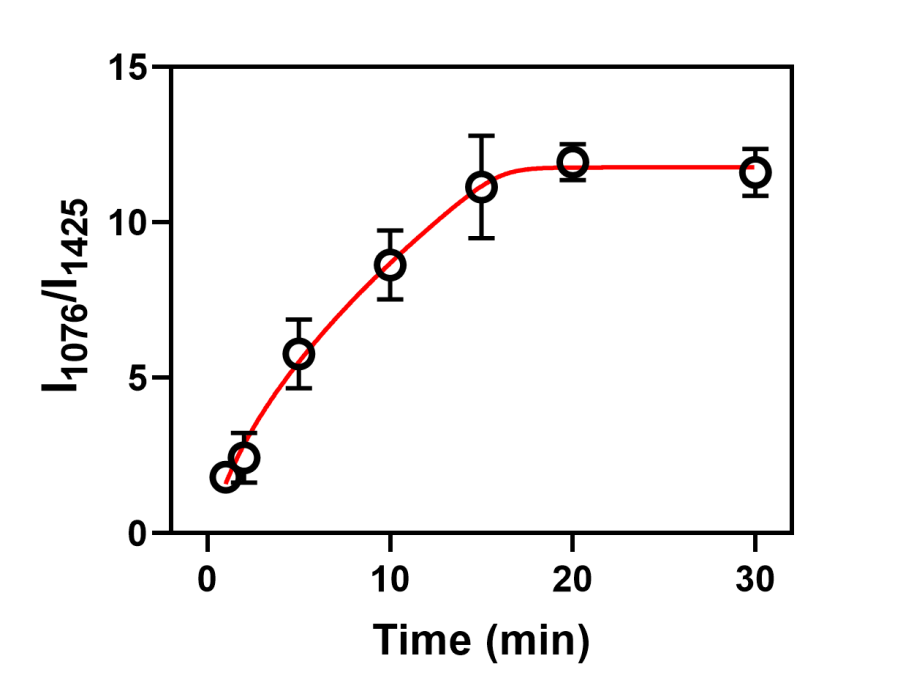


**Supplementary Figure 13.** Optimization of the reaction time of GOx for catalyzing with glucose. All data are presented as mean ± S.D. (*n* = 3).


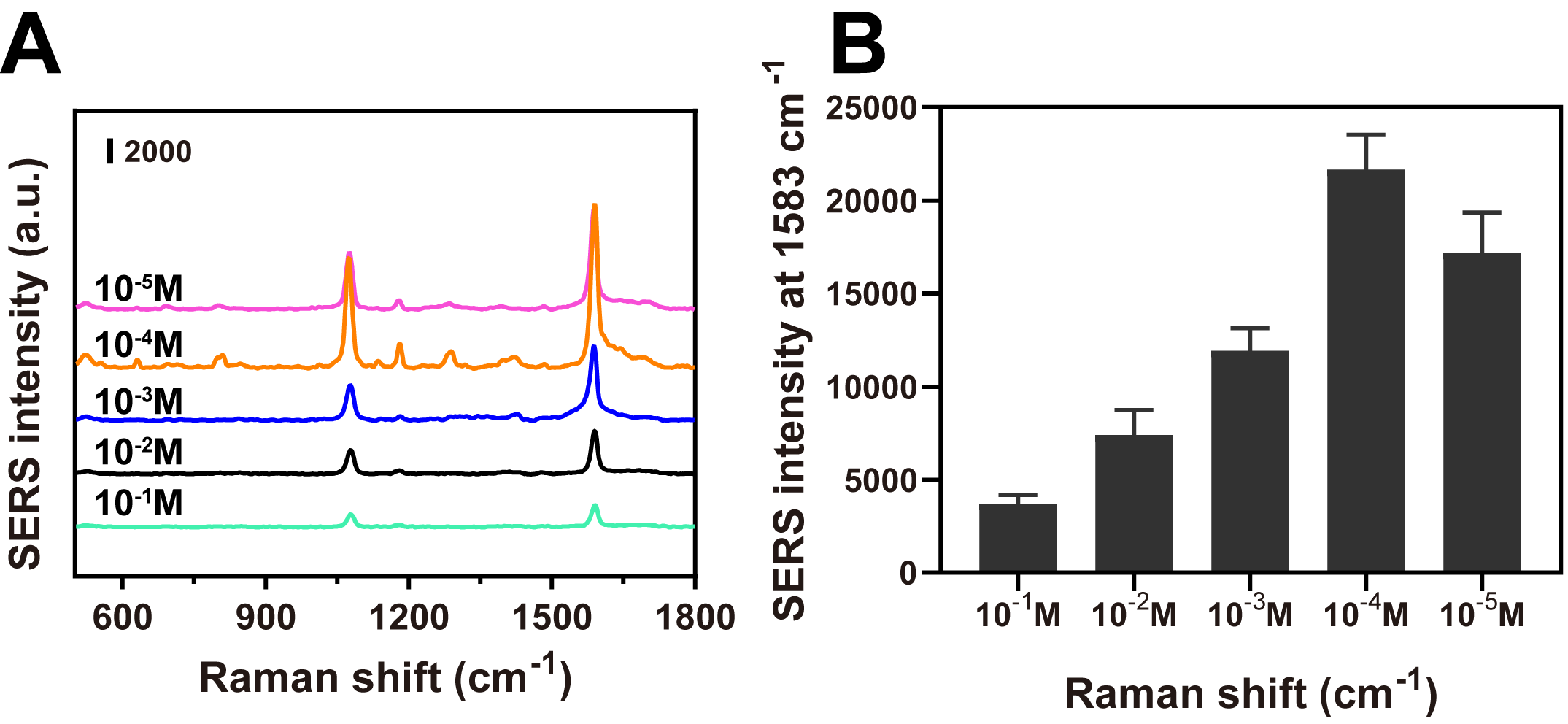


**Supplementary Figure 14.** (A) Optimization the concentration of Raman reporter 4-MBA for pH sensing. (B) SERS intensity at 1583 cm^-1^ according to (A). All data are presented as mean ± S.D. (*n* = 3).


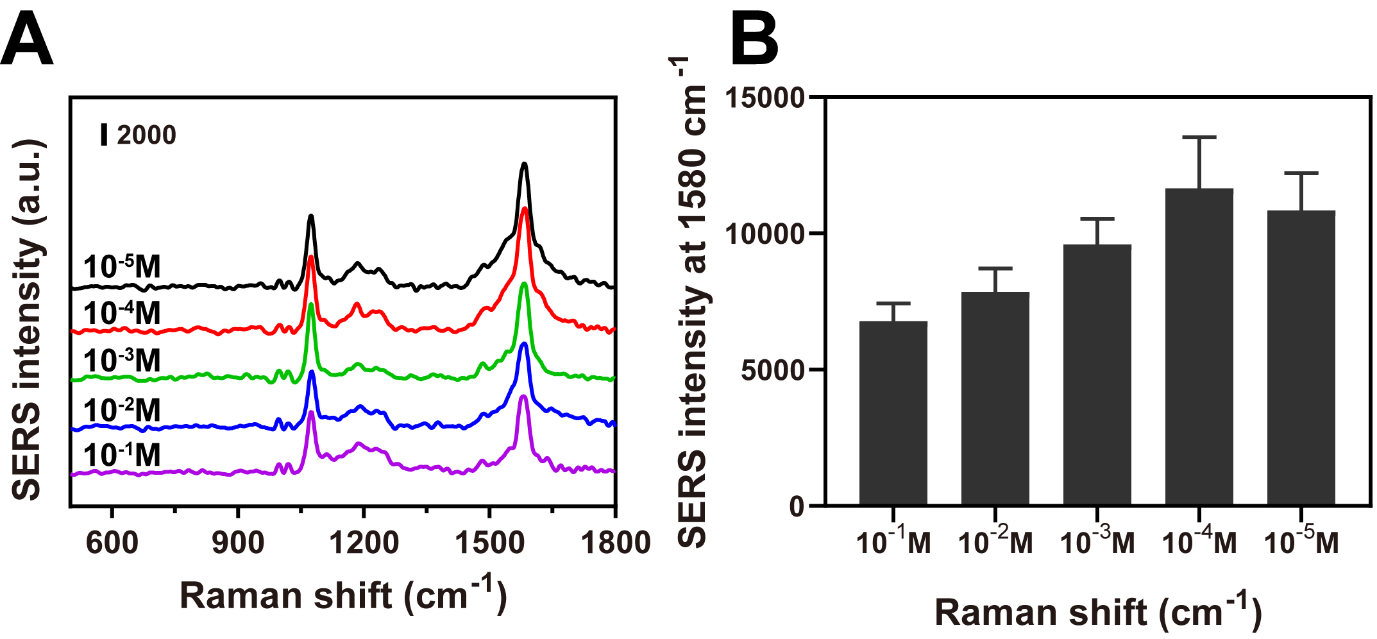


**Supplementary Figure 15.** (A) Optimization the concentration of Raman reporter 4-MPBA for glucose sensing. (B) SERS intensity at 1580 cm^-1^ according to (A). All data are presented as mean ± S.D. (*n* = 3).


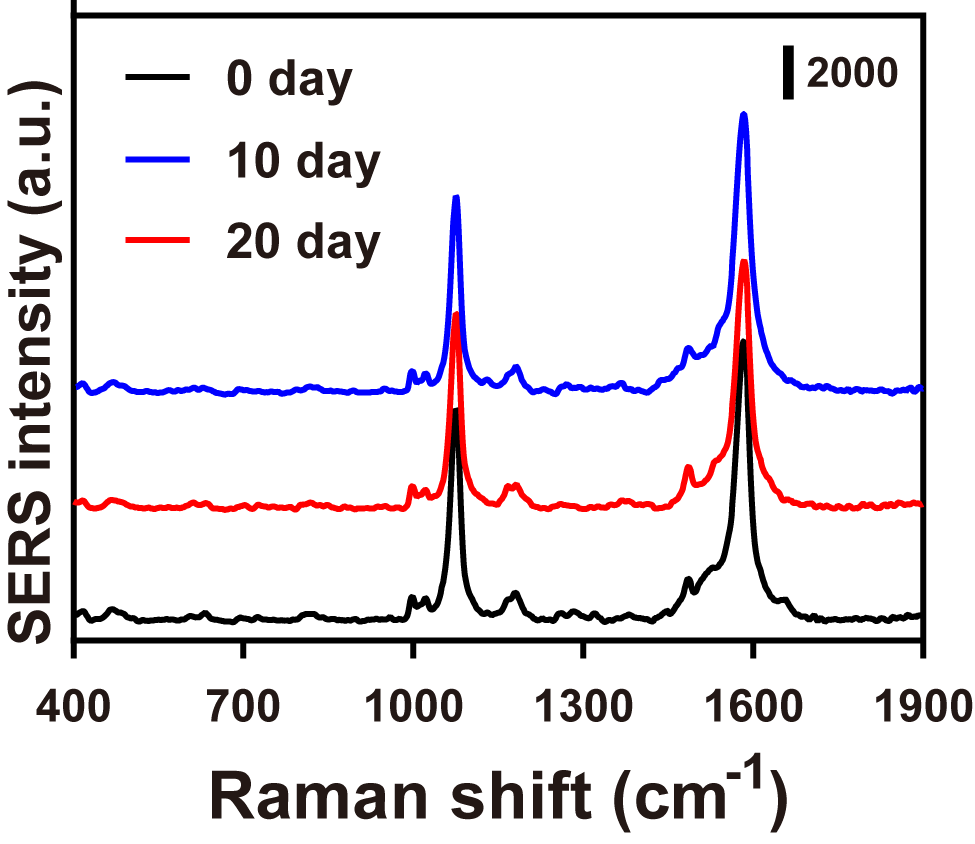


**Supplementary Figure 16.** SERS spectra of SERS-MN before and after stored at 4 °C for 0, 10 and 20 days.


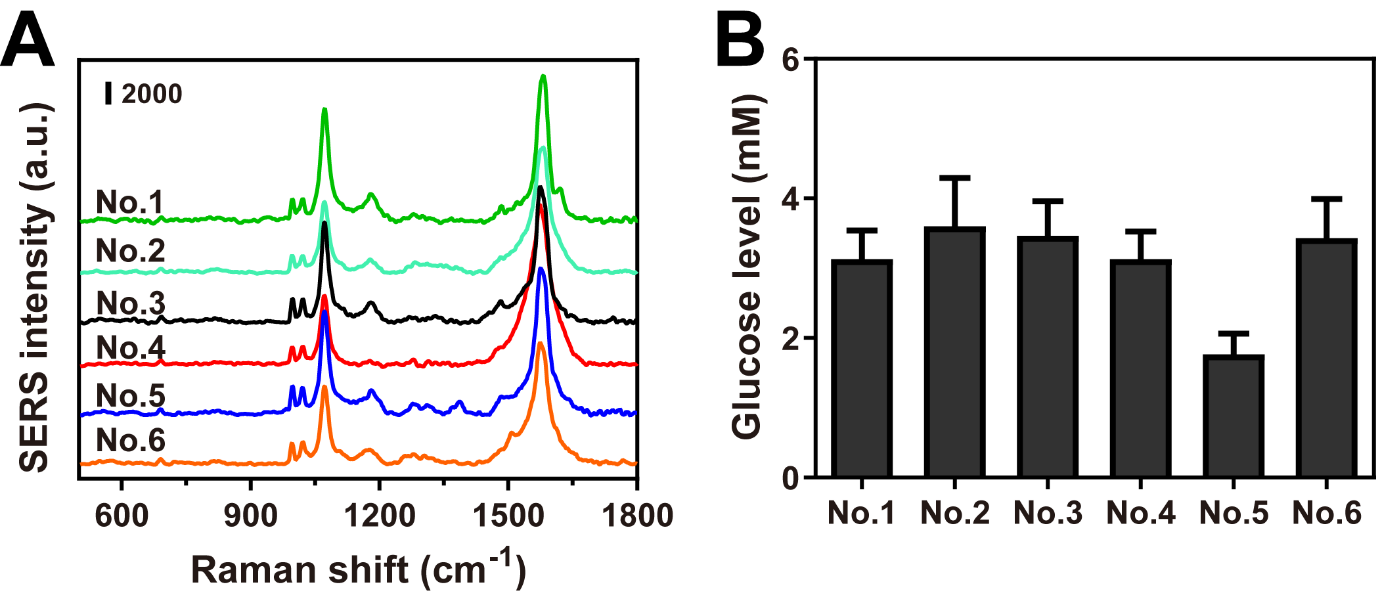


**Supplementary Figure 17.** (A) SERS response of bilayer MN array in each normal mice. (B) Quantification of ISF glucose level from (A). All data are presented as mean ± S.D. (*n* = 3).


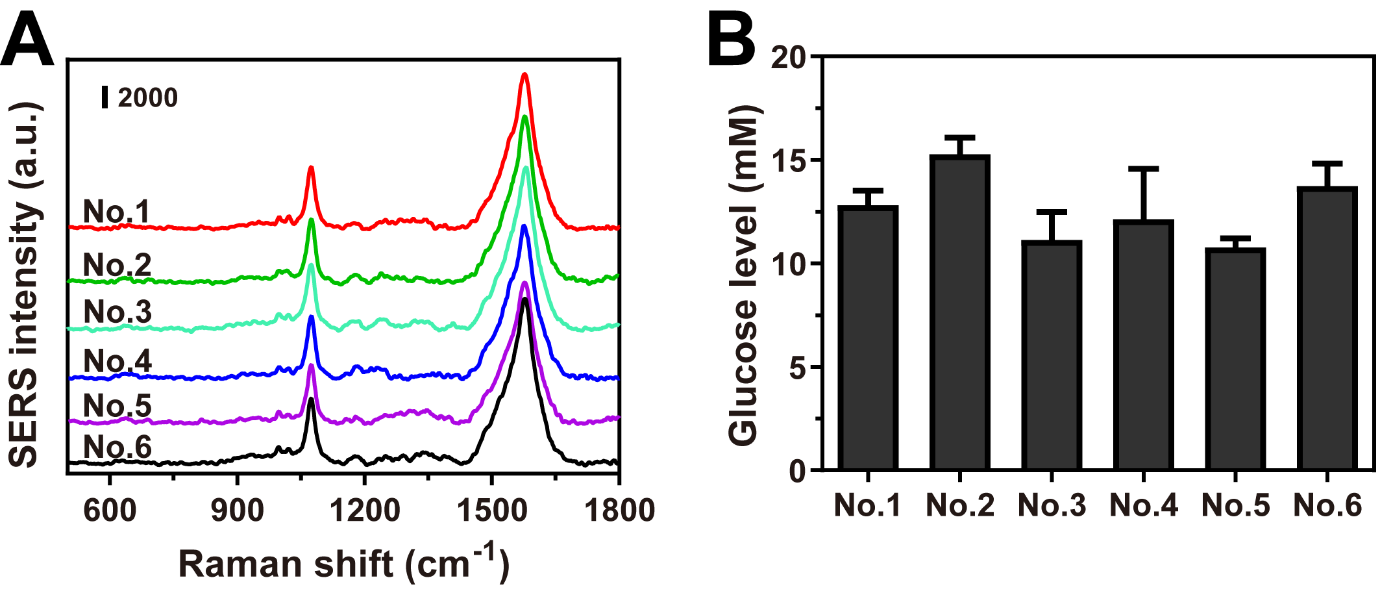


**Supplementary Figure 18.** (A) SERS response of bilayer MN array in each diabetic mice. (B) Quantification of ISF glucose level from (A). All data are presented as mean ± S.D. (*n* = 3).


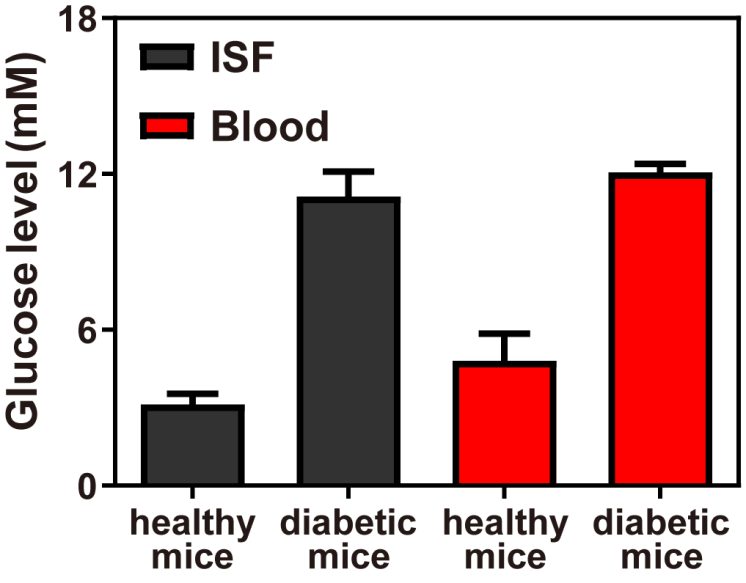


**Supplementary Figure 19.** Glucose levels of healthy and diabetic mice in blood or ISF. All data are presented as mean ± S.D. (*n* = 3).


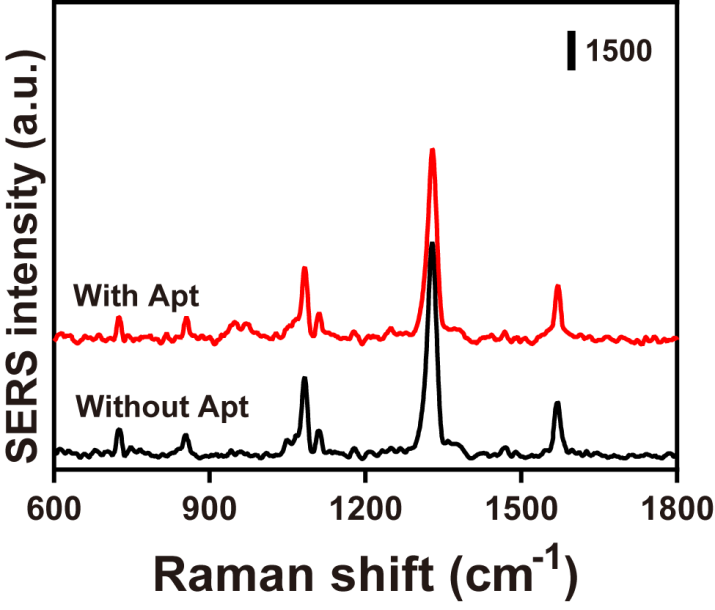


**Supplementary Figure 20.** SERS response of U@mP@Au before or after aptamer modification.


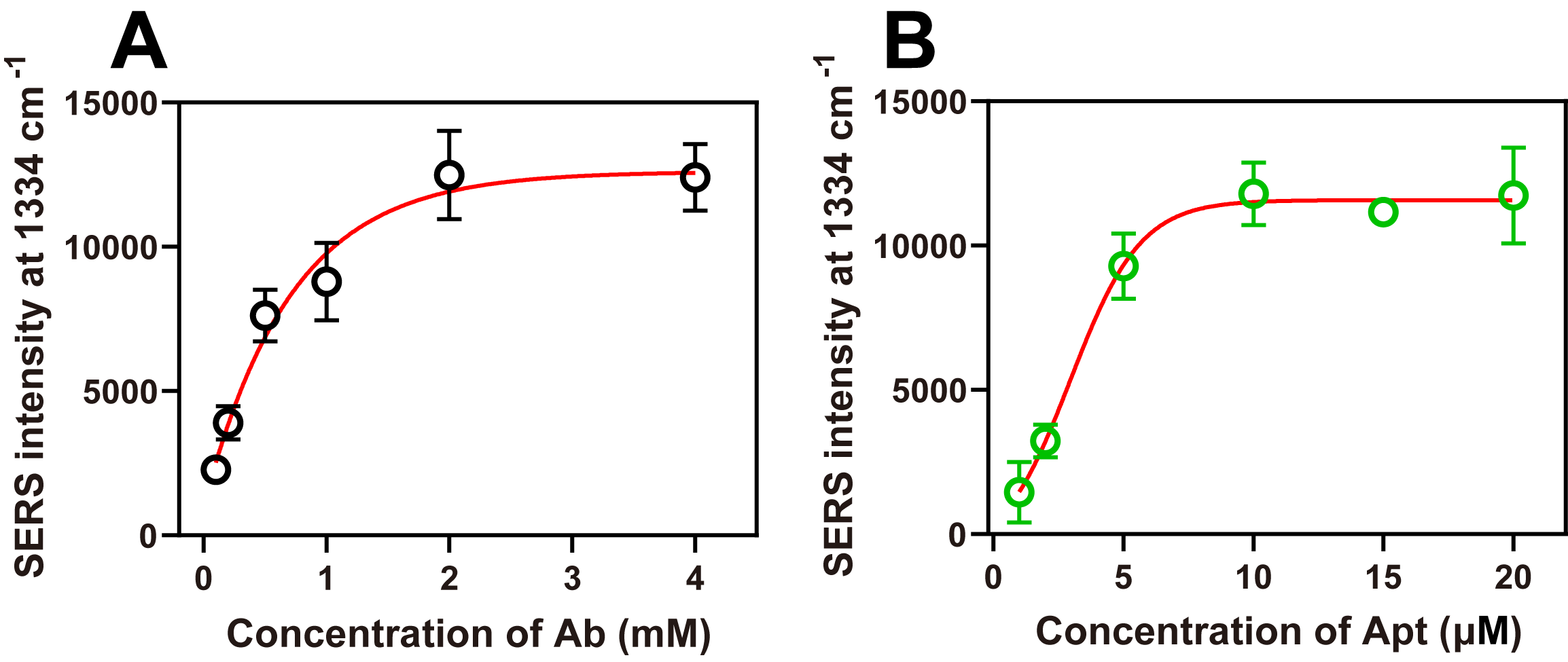


**Supplementary Figure 21.** (A) Optimization of antibody concentration. (B) Optimization of aptamer concentration. All data are presented as mean ± S.D. (*n* = 3).


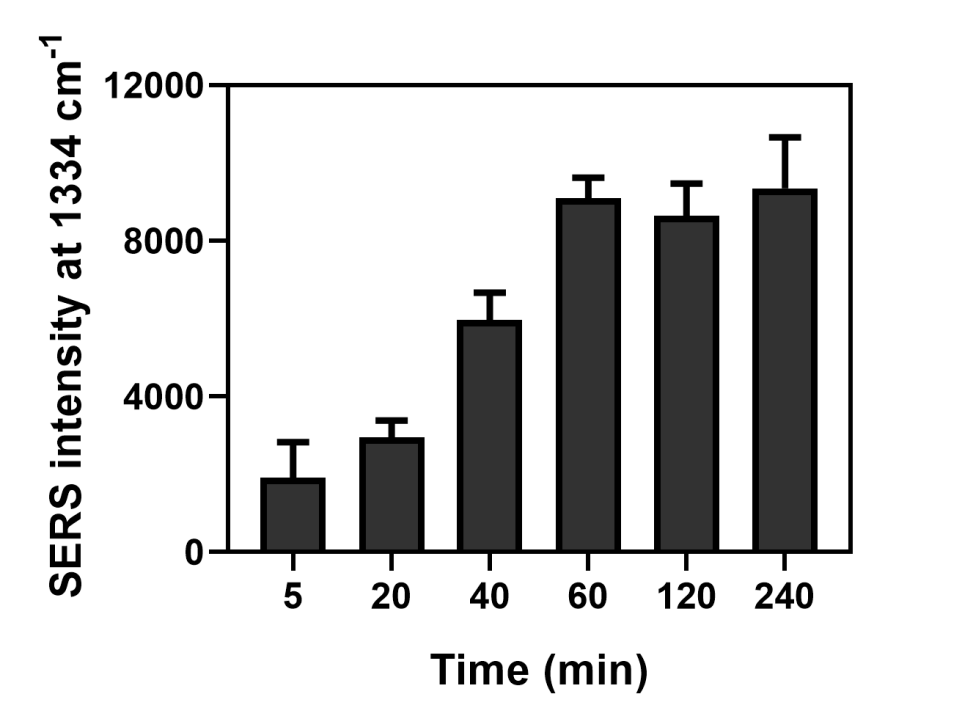


**Supplementary Figure 22.** Exploration the reaction time of U@mP@Au and ESAT-6/CFP-10. All data are presented as mean ± S.D. (*n* = 3).


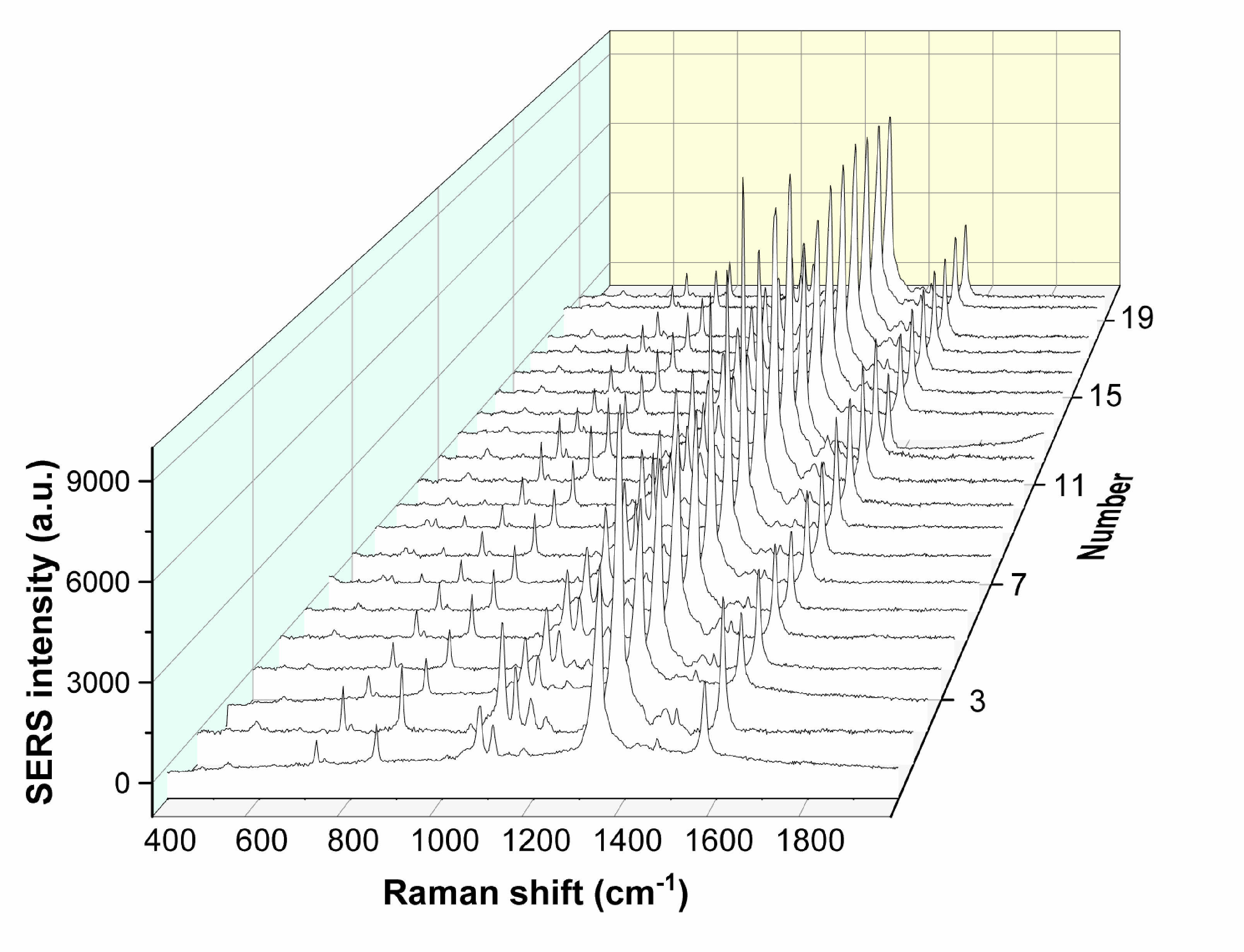


**Supplementary Figure 23.** 20 randomly selected SERS spectra acquired from the MN surface.


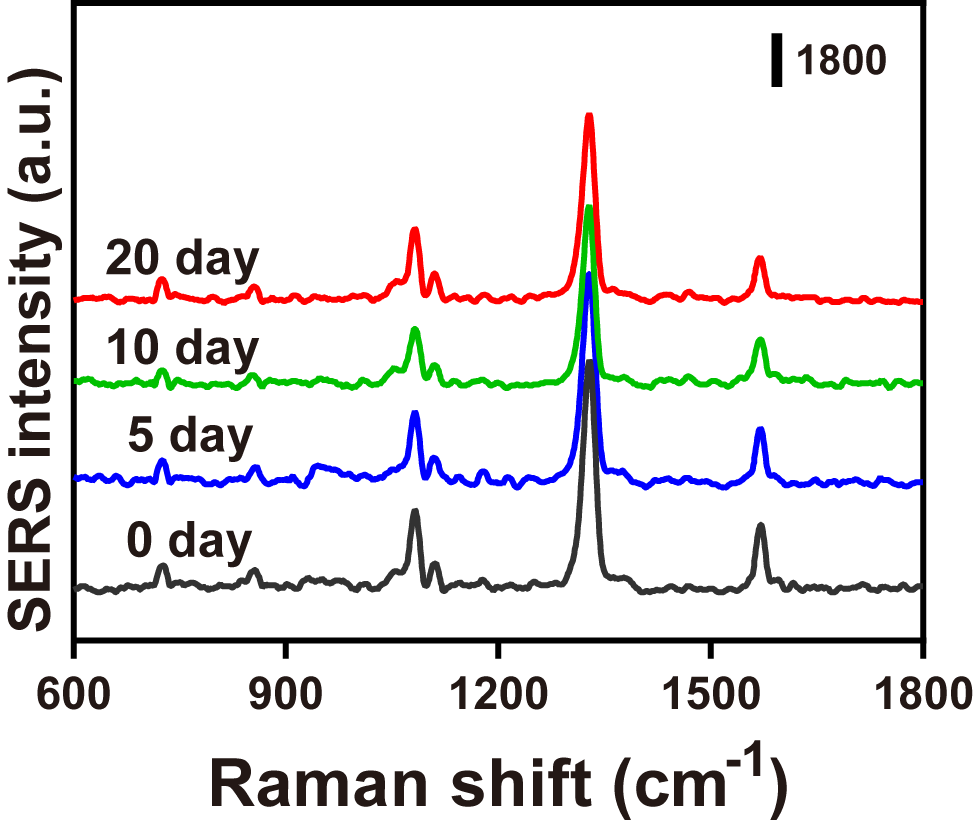


**Supplementary Figure 24.** The stability of ESAT-6/CFP-10 detection.


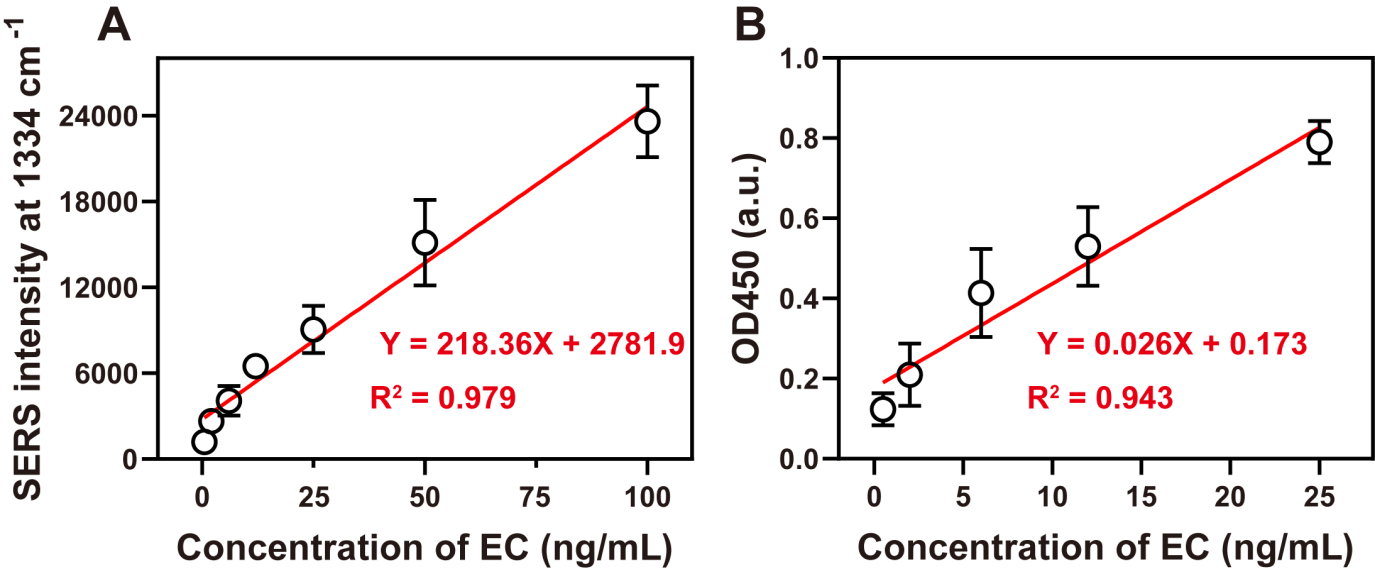


**Supplementary Figure 25.** (A) Standard curves of the SERS peak intensity at 1334 cm^-1^ against the concentrations of ESAT-6/CFP-10. (B) Standard curves of the absorption intensity at 450 nm against the concentrations of ESAT-6/CFP-10. All data are presented as mean ± S.D. (*n* = 3).


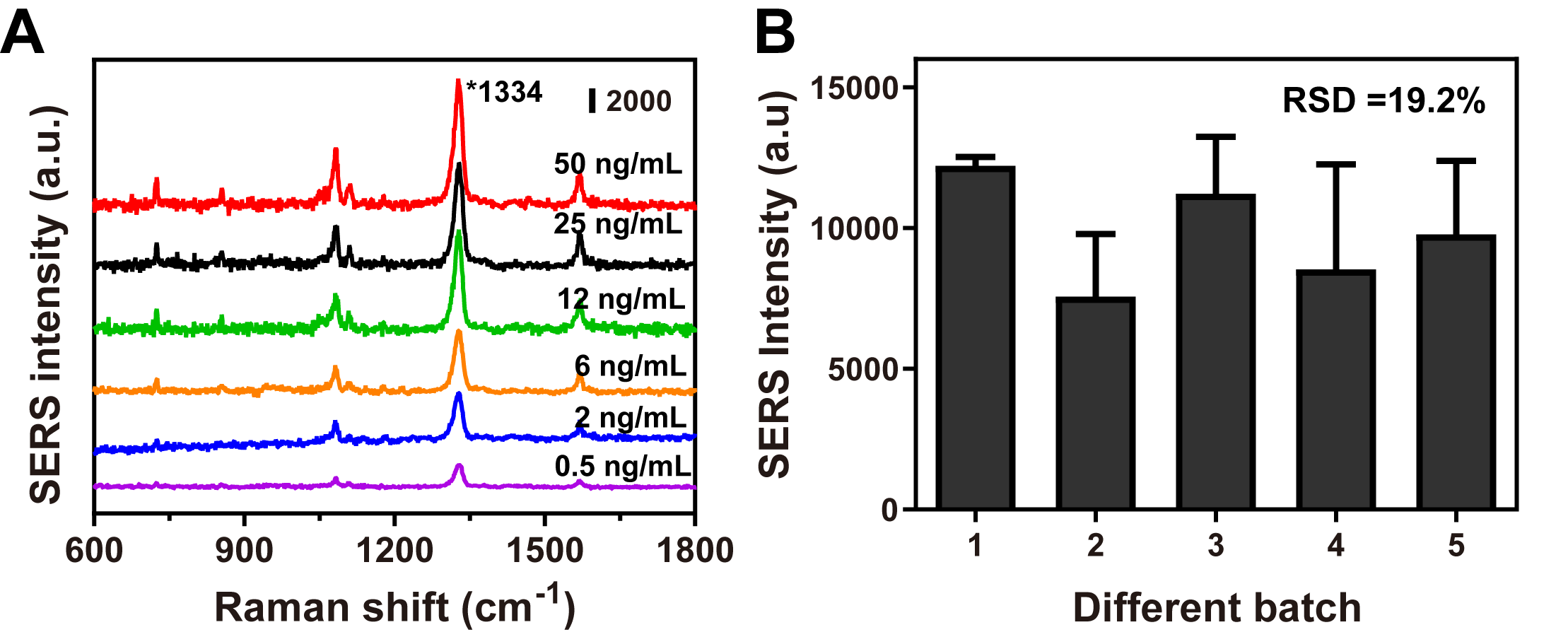


**Supplementary Figure 26.** (A) SERS spectra of SERS-MN system detected for various concentrations of ESAT-6/CFP-10 under portable Raman spectrometer. (B) Reproducibility of results recorded by the portable Raman spectrometer (ESAT-6/CFP-10 = 50 ng/mL). All data are presented as mean ± S.D. (*n* = 3).


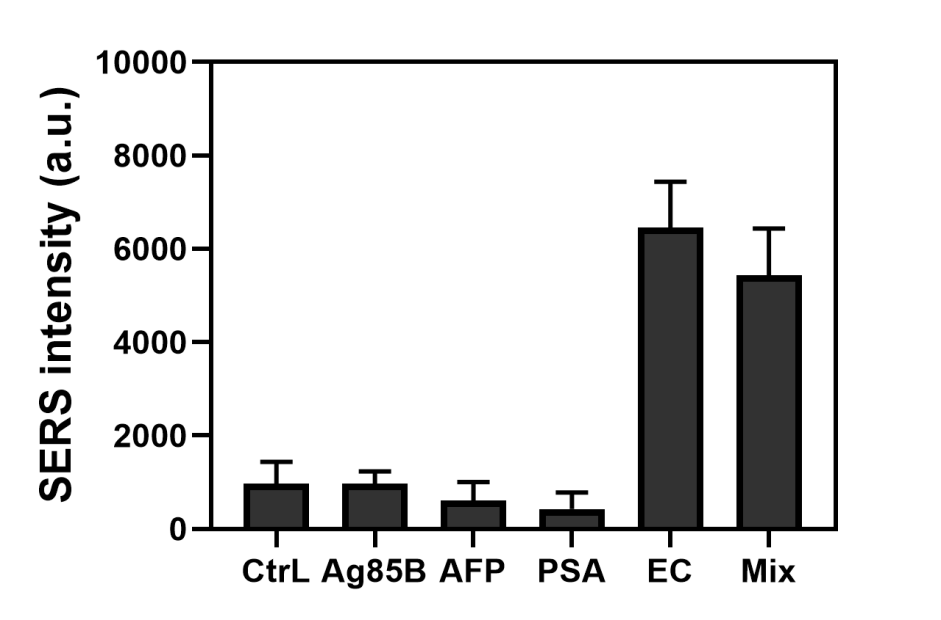


**Supplementary Figure 27.** Specificity evaluation of this immunized SERS-MN for interfering proteins and mixture. Mix = ESAT-6/CFP-10 + Ag85 + AFP + PSA. All data are presented as mean ± S.D. (*n* = 6).


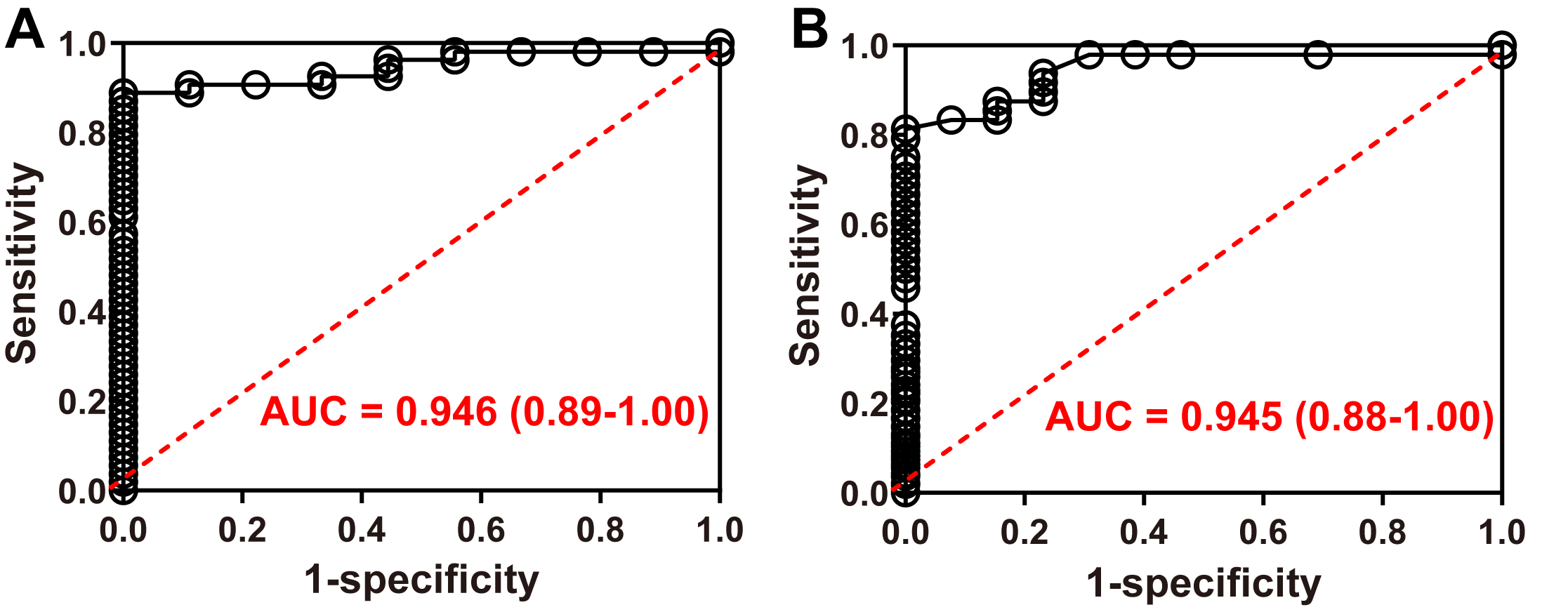


**Supplementary Figure 28.** (A) ROC curve of SERS-MN for DM diagnosis. Area under the ROC curve is 0.946, 95% CI (0.89-1.00), sensitivity is 88.9%, and specificity is 100%. (B) ROC curve of SERS-MN for TB diagnosis. Area under the ROC curve is 0.945, 95% CI (0.88-1.00), sensitivity is 87.5%, and specificity is 90.9%.

**Supplementary Table 1. Comparison of MN in different methods**

| Platform | Model | Pproperty | Application | Advantage | Reference |
| --- | --- | --- | --- | --- | --- |
| MNP-based SenBox | colorimetry | robustness, non-flexible | IgA and S1P | saliva and sputum co-detection, rapid (30 min) | [1] |
| Multipoint microneedle pH sensor | electrochemistry | mechanical stability, scalability | pH | conformable, wearable, *in situ* test | [2] |
| Plasmonic fluor immunosorbent microneedle patch | fluorescence | robustness, non-flexible | protein biomarkers | ultrasensitive and high efficient, easy to operate | [3] |
| IS-SERS-MNs | SERS | robustness, non-flexible | bacterial metabolites | highly sensitive, reliable, low cost, fast | [4] |
| Aptamer-decorated porous microneedles | fluorescence | robustness, non-flexible | endotoxin | highly sensitive, rapid, specific | [5] |
| Programmed DNA hydrogels assembled microneedles | fluorescence | robustness, non-flexible | miRNA | cascade amplification, real-time monitoring | [6] |
| wearable continuous immunoassay-based monitoring microneedles | colorimetry | robustness, non-flexible | C-peptide | real-time reading, repeatable, wearable | [7] |
| 3D printed biocompatible microneedles | electrochemistry | robustness, scalability | glucose, lactate, and alcohol | multi-day monitoring, biocompatible, low cost | [8] |
| Bilateral SERS-microneedle patch | SERS | robustness, scalability | glucose, pH, ESAT-6/CFP-10 | co-diagnosis of TB-DM comorbidity, flexible, highly sensitive | This work |

**Supplementary Table 2. Clinical background of DM patients.**

| Sample  no | Sex | age | glucometer | |
| --- | --- | --- | --- | --- |
|  |  |  | glucose  (mM) | SD |
| H1 | M | 36 | 3.5 | 2.1 |
| H2 | F | 38 | 4.5 | 1.3 |
| H3 | F | 45 | 4.3 | 0.8 |
| P1 | M | 42 | 7.8 | 1.6 |
| P2 | M | *N.A.* | 7.4 | 0.9 |
| P3 | M | 62 | 7.5 | 0.8 |
| P4 | F | *N.A.* | 7.2 | 1.0 |
| P5 | F | *N.A.* | 8.1 | 1.6 |
| P6 | M | 58 | 6.4 | 1.7 |
| P7 | M | 55 | 6.2 | 1.4 |
| P8 | F | 31 | 12.8 | 2.9 |
| P9 | M | 29 | 13.1 | 3.4 |
| P10 | M | 51 | 13.0 | 2.5 |
| P11 | M | 47 | 17.5 | 1.3 |
| P12 | M | 36 | 9.7 | 2.1 |
| P13 | F | 55 | 8.8 | 2.8 |
| P14 | M | *N.A.* | 7.3 | 1.1 |
| P15 | F | 67 | 12.2 | 0.8 |
| P16 | F | 33 | 5. 7 | 0.7 |
| P17 | M | 36 | 10. 7 | 4.0 |
| P18 | M | 45 | 18 | 2 |

Notice: *N.A.*= not applicable.

**Supplementary Table 3. Clinical background of TB patients.**

| Sample  no | Sex | age | DM comorbidity | Other information |
| --- | --- | --- | --- | --- |
|  |  |  |  |  |
| P1 | M | 36 | × | *N.A.* |
| P2 | F | 47 | × | Stenotrophomonas maltophilia/Candida albicans |
| P3 | M | 32 | × | Syphilis/  Klebsiella pneumoniae |
| P4 | M | 28 | × | *N.A.* |
| P5 | F | 54 | × | *N.A.* |
| P6 | M | 30 | × | *N.A.* |
| P7 | M | 54 | × | Syphilis |
| P8 | F | 53 | × | *N.A.* |
| P9 | F | *N.A.* | √ | Syphilis/  Streptococcus pneumoniae |
| P10 | F | 23 | × | *N.A.* |
| P11 | F | 37 | × | *N.A.* |
| P12 | M | 70 | × | *N.A.* |
| P13 | M | 36 | × | *N.A.* |
| P14 | F | 61 | × | *N.A.* |
| P15 | M | 50 | √ | Candida albicans/Staphylococcus aureus/Streptococcus pneumoniae |
| P16 | F | 68 | × | *N.A.* |
| P17 | F | 55 | × | Syphilis |
| P18 | F | *N.A.* | × | *N.A.* |
| P19 | M | *N.A.* | × | *N.A.* |
| P20 | F | *N.A.* | √ | *N.A.* |
| P21 | M | 33 | × | *N.A.* |
| P22 | M | *N.A.* | × | *N.A.* |
| P23 | F | 37 | *N.A* | *N.A.* |
| P24 | F | 42 | *N.A* | *N.A.* |
| P25 | M | 59 | *N.A.* | *N.A.* |
| P26 | M | 52 | *N.A* | *N.A.* |
| P27 | M | 32 | *N.A* | *N.A.* |
| P28 | F | 41 | *N.A* | *N.A.* |
| P29 | F | 65 | *N.A* | *N.A.* |
| P30 | M | 58 | *N.A.* | *N.A.* |
| P31 | F | 39 | *N.A.* | *N.A.* |
| P32 | M | 42 | *N.A.* | *N.A.* |
| P33 | F | 37 | *N.A.* | *N.A.* |

Notice: *N.A.*= not applicable.

**References**

[1] Y.-P. Hsu, N.-S. Li, H.-H. Pang, Y.-C. Pan, H.-P. Tsai, H.-C. Chen, Y.-T. Chen, C.-H. Weng, S.-W. Kuo, H.-W. Yang, Lab-on-the-Needles: A Microneedle PatchBased Mobile Unit for Highly Sensitive Ex Vivo and In Vivo Detection of Protein Biomarkers, *ACS Nano* **2025**, *19*, 3249.

[2] W. Lee, S.-H. Jeong, Y.-W. Lim, H. Lee, J. Kang, H. Lee, I. Lee, H.-S. Han, S. Kobayashi, M. Tanaka, B.-S. Bae, Conformable microneedle pH sensors via the integration of two different siloxane polymers for mapping peripheral artery disease, *Sci Adv* **2021**, *7(48)*, eabi6290.

[3] Z. Wang, J.Luan, A. Seth, L. Liu, M. You, P. Gupta, P. Rathi, Y. Wang, S. Cao, Q. Jiang, X. Zhang, R.Gupta, Q. Zhou, J. J Morrissey, E. L Scheller, J. S Rudra, S. Singamaneni, Microneedle patch for the ultrasensitive quantification of protein biomarkers in interstitial fluid, *Nat Biomed Eng* **2021**, *5(1)*, 64.

[4] R. Mei, Y. Wang, S. Shi, X. Zhao, Z. Zhang, X. Wang, D. Shen, Q. Kang, L. Chen, Highly Sensitive and Reliable Internal-Standard Surface-Enhanced Raman Scattering Microneedles for Determination of Bacterial Metabolites as Infection Biomarkers in Skin Interstitial Fluid, *Anal Chem* **2022**, *94*, 16069.

[5] K. Yi, Y. Wang, K. Shi, J. Chi, J. Lyu, Y. Zhao, Aptamer-decorated porous microneedles arrays for extraction and detection of skin interstitial fluid biomarkers, *Biosens Bioelectron* **2021**, *190*, 113404.

[6] Q. Yang, Y. Wang, T. Liu, C. Wu, J. Li, J. Cheng, W. Wei, F. Yang, L. Zhou, Y. Zhang, S. Yang, H. Dong, Microneedle Array Encapsulated with Programmed DNA Hydrogels for Rapidly Sampling and Sensitively Sensing of Specific MicroRNA in Dermal Interstitial Fluid, *ACS Nano* **2022**, *16*, 18366.

[7] S. Chen, Z. Guo, B. Lu, M. Sun, S. Wang, S. Li, Y. Jiang, Q. Wei, D. Wang, X. Jiang, A wearable device for continuous immunoassay-based monitoring of C-peptide in interstitial fluid, *Sci Adv* 2025, 11(29), eadw2182.

[8] G. Zhong, Q. Liu, Q. Wang, H. Qiu, H. Li, T. Xu, Fully integrated microneedle biosensor array for wearable multiplexed fitness biomarkers monitoring, *Biosens Bioelectron* **2024**, *265*, 116697.
